# Insulin-like growth factor 1 modulates bioengineered tooth morphogenesis

**DOI:** 10.1038/s41598-018-36863-6

**Published:** 2019-01-23

**Authors:** Toshihito Oyanagi, Nobuo Takeshita, Mamiko Hara, Etsuko Ikeda, Toko Chida, Daisuke Seki, Michiko Yoshida, Masahiro Seiryu, Ikuko Takano, Seiji Kimura, Masamitsu Oshima, Takashi Tsuji, Teruko Takano-Yamamoto

**Affiliations:** 1 Division of Orthodontics and Dentofacial Orthopedics, Graduate School of Dentistry, Tohoku University, Sendai, Miyagi 980-0875 Japan; 2 Laboratory for Organ Regeneration, RIKEN Center for Developmental Biology, Kobe, Hyogo 650-0047 Japan; 3 Department of Stomatognathic Function and Occlusal Reconstruction, Graduate School of Biomedical Sciences, Tokushima University, Tokushima, Tokushima 770-8501 Japan; 4 Department of Biomaterials and Bioengineering, Faculty of Dental Medicine, Hokkaido University, Sapporo, Hokkaido 060-8586 Japan

## Abstract

Regenerative therapy to replace missing teeth is a critical area of research. Functional bioengineered teeth have been produced by the organ germ method using mouse tooth germ cells. However, these bioengineered teeth are significantly smaller in size and exhibit an abnormal crown shape when compared with natural teeth. The proper sizes and shapes of teeth contribute to their normal function. Therefore, a method is needed to control the morphology of bioengineered teeth. Here, we investigated whether insulin-like growth factor 1 (IGF1) can regulate the sizes and shapes of bioengineered teeth, and assessed underlying mechanisms of such regulation. IGF1 treatment significantly increased the size of bioengineered tooth germs, while preserving normal tooth histology. IGF1-treated bioengineered teeth, which were developed from bioengineered tooth germs in subrenal capsules and jawbones, showed increased sizes and cusp numbers. IGF1 increased the number of *fibroblast growth factor* (*Fgf4*)-expressing enamel knots in bioengineered tooth germs and enhanced the proliferation and differentiation of dental epithelial and mesenchymal cells. This study is the first to reveal that IGF1 increases the sizes and cusp numbers of bioengineered teeth via the induction of enamel knot formation, as well as the proliferation and differentiation of dental epithelial and mesenchymal cells.

## Introduction

In the field of tooth regeneration, the ultimate goal is the regeneration of fully functional teeth in living bodies^1^. Tsuji’s group developed the organ germ method, which is a three-dimensional cell manipulation technique for the generation of bioengineered tooth germs^2^. Upon transplantation into the jawbones of mice, bioengineered tooth germs developed into bioengineered teeth and erupted into the oral cavity; these bioengineered teeth exhibited similar hardness to that of natural teeth, and were responsive to noxious stimuli^3^. These reports suggest that functional bioengineered teeth can be regenerated via transplantation of *in vitro*-generated bioengineered tooth germs into an edentulous area of the jaw.

The proper sizes and shapes of organs contribute to their normal function. There are site-specific types of teeth in the dentition, such as incisors, canines and molars. These have different sizes and shapes, and play unique roles in stomatognathic function and aesthetics. Tooth size reportedly affects the rate of food breakdown^4^; furthermore, tooth shape correlates with masticatory efficiency^5^. From a clinical perspective, each patient exhibits individual sizes and shapes of teeth that are genetically and environmentally determined. Accordingly, the goal of dental regenerative therapy is to generate bioengineered teeth that erupt into the oral cavity, with sizes and shapes similar to those of natural teeth, in order to rehabilitate the aesthetics and stomatognathic function of individual patients. However, the bioengineered mouse teeth generated by Tsuji’s group were remarkably small in size and exhibited an abnormal crown shape, compared with that of natural teeth^3^. Therefore, a method is needed to control the morphology of bioengineered teeth.

Tooth development progresses via interactions between dental epithelial and mesenchymal tissues^1,6^. Various molecules participate in spatio-temporal regulation of cellular processes, including proliferation and differentiation, during tooth development^1,6^. Notably, molecules that affect tooth size and shape during tooth development could be useful for the morphological control of bioengineered teeth. Insulin-like growth factor 1 (IGF1) regulates the normal development of various tissues, such as bone, muscle, and nerve, by regulating cell proliferation, differentiation, apoptosis, and migration^7–9^. Leprechaunism is a congenital syndrome associated with insulin resistance; it is characterized by retarded intrauterine and postnatal growth^10^. We previously reported a leprechaunism patient who had undergone long-term treatment with recombinant IGF1 and had developed extremely large teeth^11^. Conversely, Sarnat *et al*.^12^ reported that patients with Laron-type dwarfism, which is characterized by the loss of growth hormone receptors, exhibit a deficiency in IGF1 secretion, such that they develop teeth with reduced mesiodistal width. Moreover, IGF1 and its receptor are expressed in both dental epithelial and mesenchymal tissues in tooth germs^13,14^; IGF1 signalling promotes cell proliferation, differentiation, and matrix secretion in mouse tooth germs^15,16^. Based on this previous evidence, we hypothesized that IGF1 regulates tooth morphogenesis, and can thus be used to control the size and shape of bioengineered teeth.

The aim of this study was to clarify whether IGF1 has the potential to control the size and shape of bioengineered teeth. We transplanted bioengineered tooth germs, which were treated with IGF1 during organ culture, into the jawbones of living mice, in accordance with the method of our previous report^3^. The sizes and shapes of the developed bioengineered teeth were then examined. We further analysed the effects of IGF1 on enamel knot formation, as well as on the proliferation and differentiation of dental epithelial and mesenchymal cells *in vitro*, in order to elucidate the underlying biological mechanisms that cause morphological changes in IGF1-treated bioengineered teeth.

## Results

### IGF1 increases the size of tooth germs in culture

**Figure 1:**
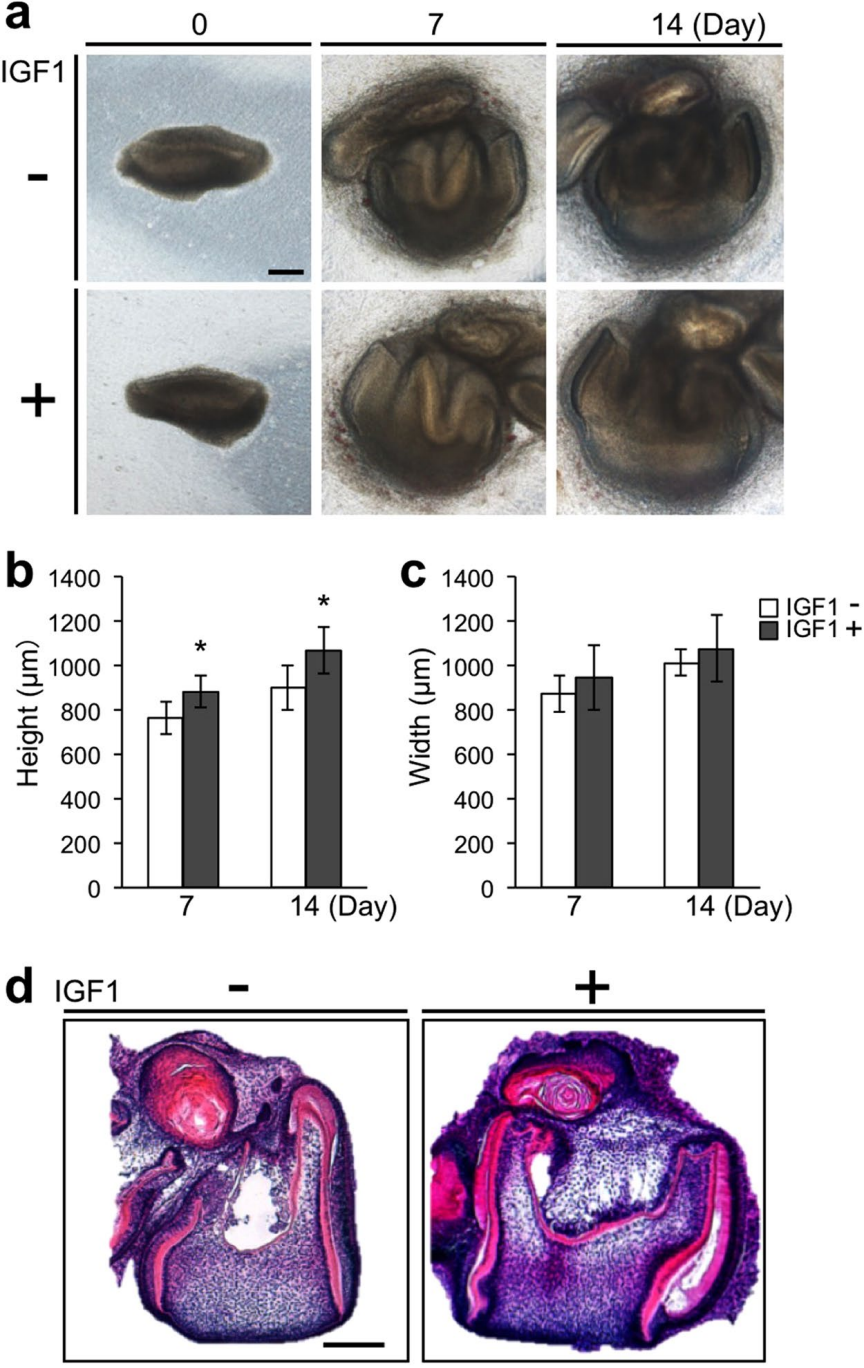
IGF1 increases the size of natural tooth germs in organ culture. (**a**) Phase-contrast images of tooth germs derived from ED14.5 mouse mandible at days 0, 7, and 14 of organ culture in the control and IGF1-treated groups. Scale bar, 200 µm. The height (**b**) and width (**c**) of tooth germs in the control and IGF1-treated groups at days 7 and 14 of organ culture. Error bars indicate the standard deviation (N = 9). *p < 0.05 (versus control group; Student’s *t*-test). (**d**) HE staining of the tooth germs at day 14 of organ culture in the control and IGF1-treated groups. Scale bar, 200 µm.

To investigate the effect of IGF1 on the morphogenesis of tooth germs in organ culture, we first treated tooth germs dissected from embryonic day (ED) 14.5 mouse mandibles with IGF1. As shown in Fig. 1a, the tooth germs exhibited increased size. Treatment with 4 µg/mL IGF1 significantly increased the height of tooth germs at days 7 and 14 (Fig. 1b). The width of IGF1-treated tooth germs became larger than that of control tooth germs at days 7 and 14 (Fig. 1c). Normal dental histological structure, comprising layers of ameloblasts, odontoblasts, enamel, and dentin, was observed in the control and IGF1-treated groups at day 14 of organ culture (Fig. 1d).

### IGF1 increases the size of bioengineered tooth germs

First, to confirm the absence of cross-contamination of dental epithelial and mesenchymal cells (the sources of cells used for the generation of the bioengineered tooth germs) during the isolation procedure, we analysed the gene expression levels of the dental epithelial markers *paired-like homeodomain transcription factor 2* (*Pitx2*), *sonic hedgehog* (*Shh*), and *fibroblast growth factor 4* (*Fgf4*), as well as the expression level of the dental mesenchymal marker *Fgf10*^17^. Expression of *Pitx2*, *Shh*, *Fgf4*, and *Fgf10* was observed in the tooth germs (Supplementary Figs S2 and S3). In dental epithelial tissues and cells, *Pitx2*, *Shh*, and *Fgf4* were expressed, whereas *Fgf10* was not (Supplementary Figs S2 and S3). In addition, *Fgf10* was expressed in dental mesenchymal tissues and cells, whereas *Pitx2*, *Shh*, and *Fgf4* were not (Supplementary Figs S2 and S3). These results indicated that there was no cross-contamination of dental epithelial and mesenchymal cells.

**Figure 2:**
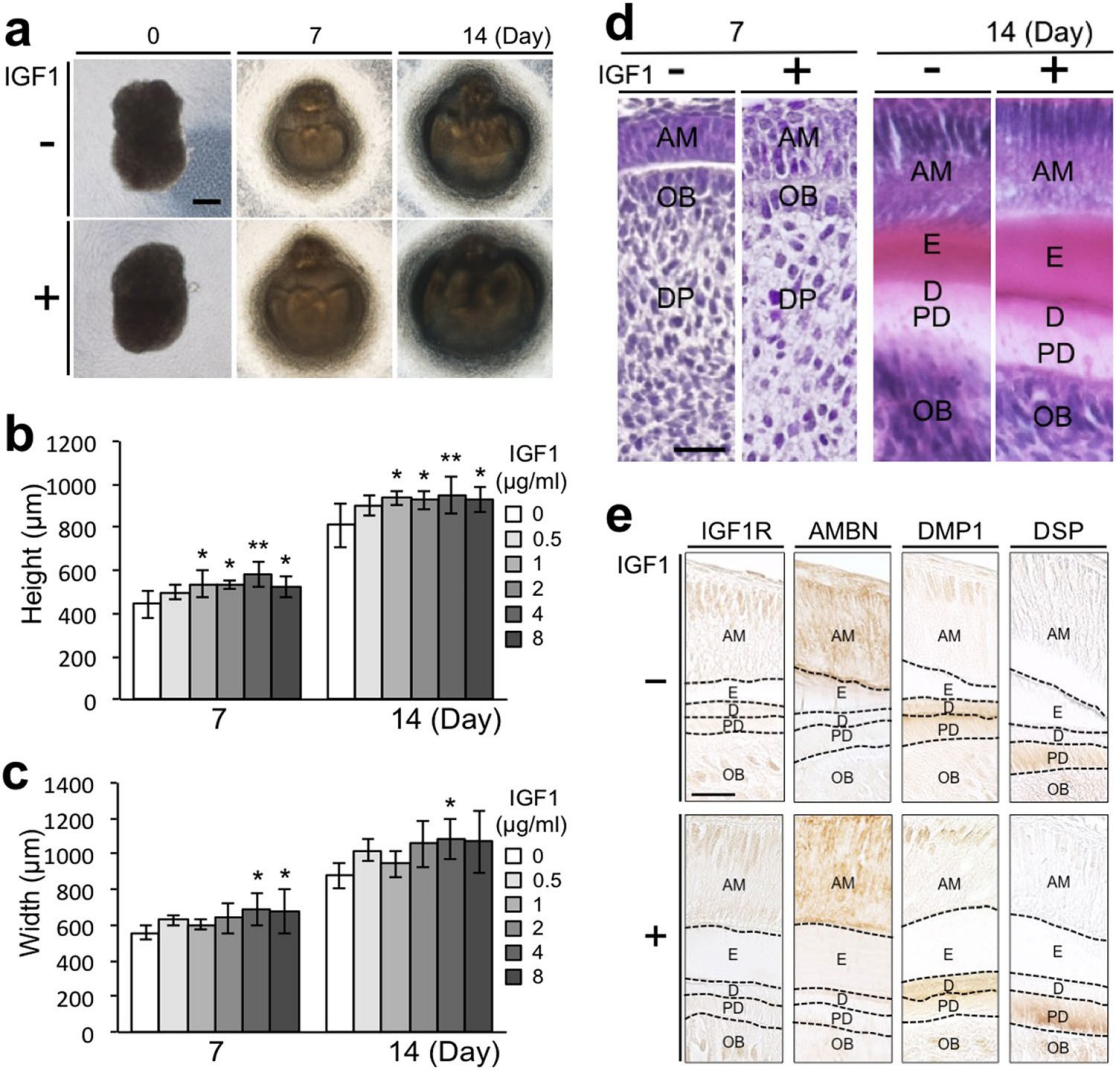
IGF1 increases the size of bioengineered tooth germs. (**a**) Phase-contrast images of tissue recombinants at day 0 and bioengineered tooth germs at days 7 and 14 of organ culture in the control and IGF1-treated groups. Scale bar, 200 µm. The height (**b**) and width (**c**) of bioengineered tooth germs at days 7 and 14 in the control and IGF1-treated groups. Error bars indicate the standard deviation (N = 6 or 7). *p < 0.05, **p < 0.01 (versus 0 µg/mL group; one-way analysis of variance followed by the Tukey-Kramer test). (**d**) HE staining of bioengineered tooth germs at days 7 and 14 in the control and IGF1-treated groups. AM, ameloblasts; D, dentin; DP, dental papilla; E, enamel; OB, odontoblasts; PD, predentin. Scale bar, 20 µm. (**e**) Immunohistochemical staining of IGF1R, AMBN, DMP1, and DSP in bioengineered tooth germs at day 14 of culture in the control and IGF1-treated groups. Scale bar, 20 µm.

The contact length between reconstituted epithelial and mesenchymal cell layers affects the size of bioengineered tooth germs generated by recombination of epithelium and mesenchyme and grown in an organ culture *in vitro* set-up^18^. Therefore, we generated tissue recombinants with a uniform contact length in the control and IGF1-treated groups. The bioengineered tooth germs formed a translucent zone between the epithelial and mesenchymal tissues at day 2, showed tooth crown shape at day 7, and grew further by day 14 (Fig. 2a, Supplementary Fig. S4). The heights and widths of the bioengineered tooth germs increased in a dose-dependent manner upon treatment with IGF1 (Fig. 2b,c). The heights of bioengineered tooth germs treated with 1, 2, 4, and 8 µg/mL IGF1 were significantly greater than the heights of the control group germs at days 7 and 14 (Fig. 2b). The widths of the bioengineered tooth germs treated with 4 µg/mL were significantly larger than those of the control group at days 7 and 14 (Fig. 2c). The widths of the bioengineered tooth germs treated with 8 µg/mL IGF1 were significantly larger than those of the control group at day 7, but not at day 14 (Fig. 2c). The maximum effects of IGF1 on the height and width of the bioengineered tooth germs were observed at 4 µg/mL. These results indicated that IGF1 increased the size of the bioengineered tooth germs.

Histological analysis of the bioengineered tooth germs in the control and IGF1-treated groups showed dental papillae, odontoblasts, and ameloblast layers at day 7, as well as deposition of enamel and dentin matrix at day 14 (Fig. 2d); this indicated no histological differences between the groups. Immunohistochemical staining demonstrated IGF1 receptor (IGF1R) expression in ameloblasts, odontoblasts and dental papilla cells in the bioengineered tooth germs in both control and IGF1-treated groups at day 7 (Supplementary Fig. S5). The expression of IGF1R was also observed in ameloblasts and odontoblasts in the bioengineered tooth germs at day 14 of culture (Fig. 2e). Ameloblastin (AMBN), an enamel matrix protein secreted by ameloblasts^19,20^, was expressed in ameloblasts; dentin matrix acidic phosphoprotein 1 (DMP1), the extracellular matrix secreted by ameloblasts and odontoblasts^21^, was expressed in ameloblasts and at the junction area between dentin and predentin; and dentin sialoprotein (DSP), a component of the dentin extracellular matrix^21^, was expressed in predentin in both groups (Fig. 2e). Thus, obvious differences in the expression patterns of these proteins were not recognized between the control and IGF1-treated groups.

### IGF1 increases the size and cusp number of bioengineered teeth developed in subrenal capsules

**Figure 3:**
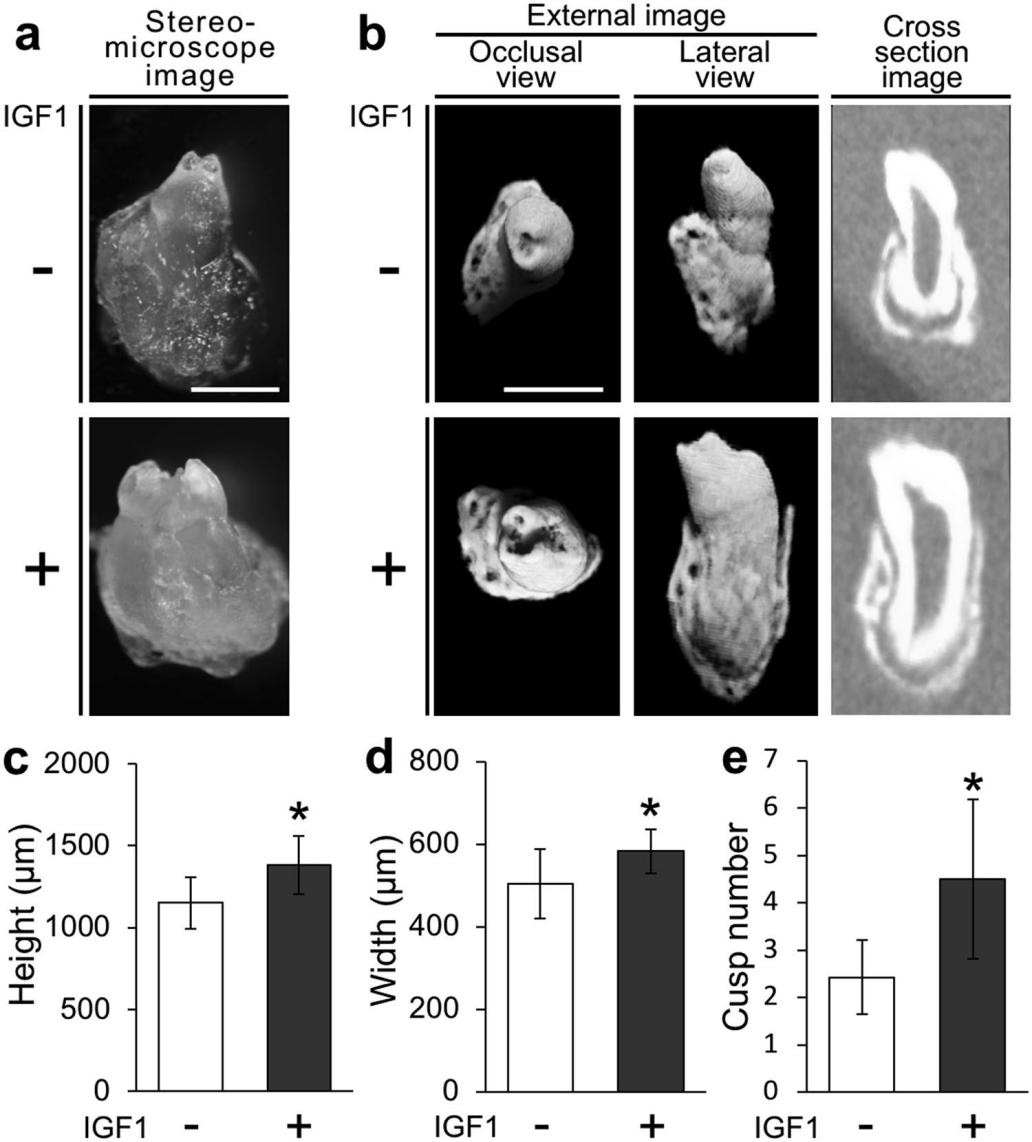
IGF1 increases the size and cusp number of bioengineered teeth developed in subrenal capsules. Stereomicroscopy images (**a**) and micro-CT images (**b**) of bioengineered teeth at 30 days after renal subcapsular transplantation in the control and IGF1-treated groups. Scale bar, 600 µm. The height (**c**), width (**d**), and cusp number (**e**) of bioengineered teeth at 30 days after renal subcapsular transplantation in the control and IGF1-treated groups. Error bars indicate the standard deviation (N = 7 or 8). *p < 0.05 (versus control group; Student’s *t*-test).

We examined the effect of IGF1 on the morphology of the bioengineered teeth. Transplantation of bioengineered tooth germs into subrenal capsules resulted in the generation of bioengineered teeth with surrounding hard tissues in the control and IGF1-treated groups (Fig. 3a,b). The height of the bioengineered teeth in the group treated with 4 µg/mL IGF1 (1379.2 ± 177.7 µm, N = 8) was significantly greater than that in the control group (1151.2 ± 156.7 µm, N = 7) (Fig. 3c). The crown width of bioengineered teeth in the group treated with 4 µg/mL IGF1(582.3 ± 54.0 µm, N = 8) was also greater than that of teeth in the control group (503.8 ± 84.6 µm, N = 7) (Fig. 3d). Cusp number is a measurable aspect of tooth shape patterning^22^. Thus, we examined the cusp number of the bioengineered teeth to analyse the effect of IGF1 on bioengineered tooth morphology. The cusp number of bioengineered teeth in the group treated with 4 µg/mL IGF1 (4.5 ± 1.7, N = 8) was greater than that of teeth in the control group (2.4 ± 0.8, N = 7) (Fig. 3e).

### IGF1-treated bioengineered teeth erupt from the jawbone with greater size and increased cusp number

**Figure 4:**
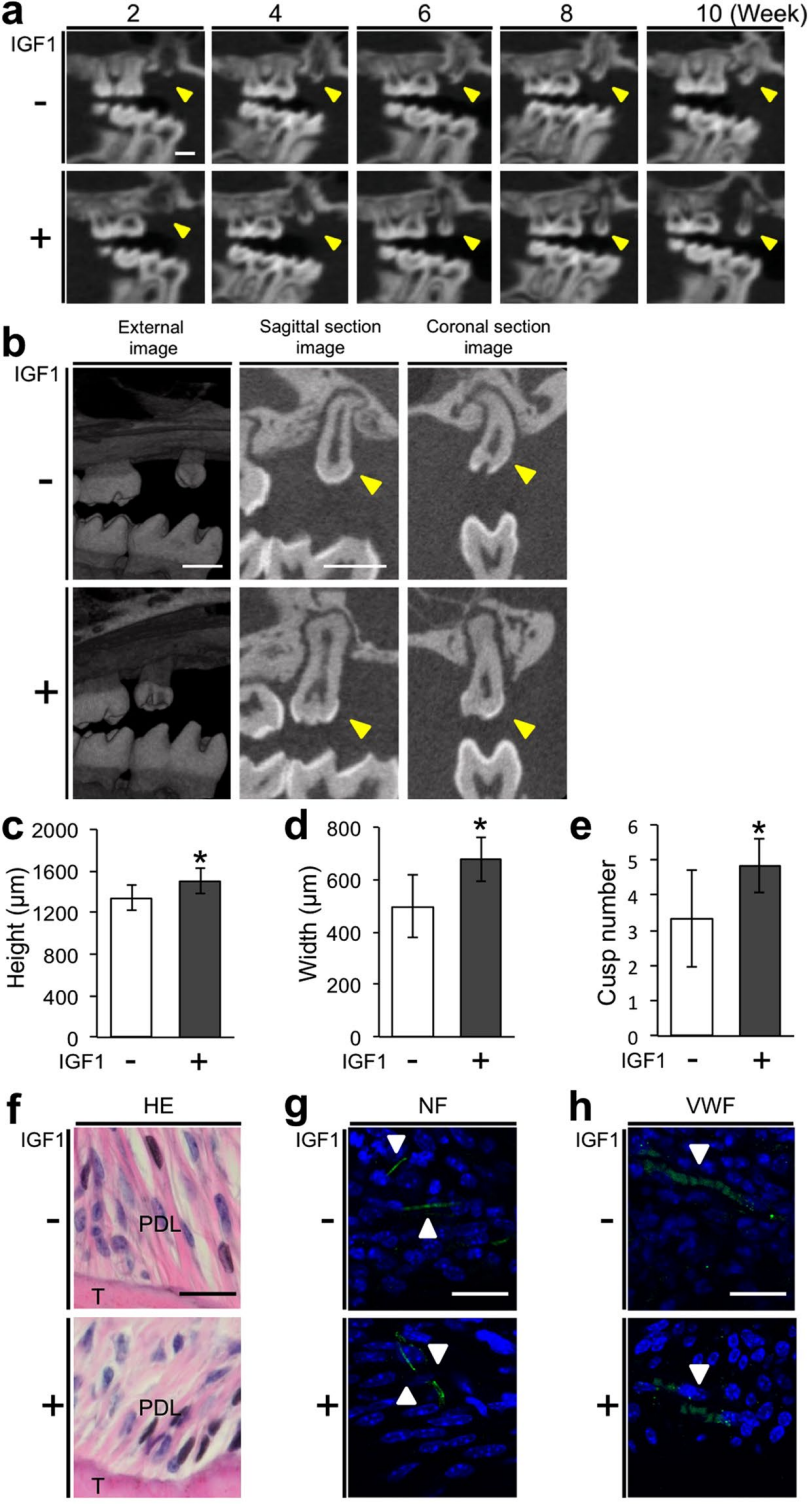
IGF1-treated bioengineered teeth erupt from the jawbone with greater size and increased cusp number. (**a**) Micro-CT images of the bioengineered tooth in the control and IGF1-treated groups at 2, 4, 6, 8, and 10 weeks after transplantation. The bioengineered teeth are indicated by arrowheads. Scale bar, 500 µm. (**b**) Micro-CT images of bioengineered teeth in the control and IGF1-treated groups at 90 days after transplantation into jawbones. The bioengineered teeth are indicated by arrowheads. Scale bar, 600 µm. The height (**c**), width (**d**), and cusp number (**e**) of bioengineered teeth at 90 days after transplantation into jawbones in the control and IGF1-treated groups. Error bars indicate the standard deviation (N = 6 or 7). *p < 0.05 (versus control group; Student’s *t*-test). Periodontal ligament surrounding the root of bioengineered teeth developed in jawbones was examined by HE staining (**f**) and immunofluorescence analysis of NF (**g**) and VWF (**h**). NF and VWF are indicated by arrowheads. Scale bar, 20 µm. PDL, periodontal ligament; T, tooth.

We further examined whether the bioengineered teeth developed from the IGF1-treated bioengineered tooth germs could erupt from a jawbone with greater size and additional cusps. Eruption of the bioengineered teeth gradually progressed over 10 weeks after transplantation in both control and IGF1-treated groups without any pathological observations, such as ankylosis or bone defects (Fig. 4a). Quantitative analysis of micro-computed tomography images indicated greater height of bioengineered teeth in the group treated with 4 µg/mL IGF1 (1500.8 ± 119.6 µm, N = 6) than in the control group (1340.6 ± 117.2 µm, N = 7) at 90 days after transplantation (Fig. 4b,c). The crown width of the bioengineered teeth in the group treated with 4 µg/mL IGF1 (677.5 ± 83.8 µm, N = 6) was also significantly greater than that of teeth in the control group (497.9 ± 118.3 µm, N = 7) (Fig. 4b,d). Furthermore, the cusp number of bioengineered teeth in the group treated with 4 µg/mL IGF1 (4.8 ± 0.8, N = 6) was greater than that of teeth in the control group (3.3 ± 1.4, N = 7) (Fig. 4e).

Periodontal ligament (PDL) is a fibrous tissue located between the tooth root and alveolar bone^23^. PDL surrounding the bioengineered tooth root was observed in the control and IGF1-treated groups (Fig. 4f). IGF1 did not visibly affect the histology of the PDL (Fig. 4f). We investigated the expression of the nerve fibre marker, neurofilament (NF), and the vascular endothelial cell marker, von Willebrand factor (VWF), in the PDL. NF and VWF were expressed in the PDL in both control and IGF1-treated groups. There were no clear differences in the expression patterns of NF and VWF in the PDL surrounding control and IGF1-treated bioengineered teeth (Fig. 4g,h).

### IGF1 does not affect the hardness of bioengineered teeth

**Figure 5:**
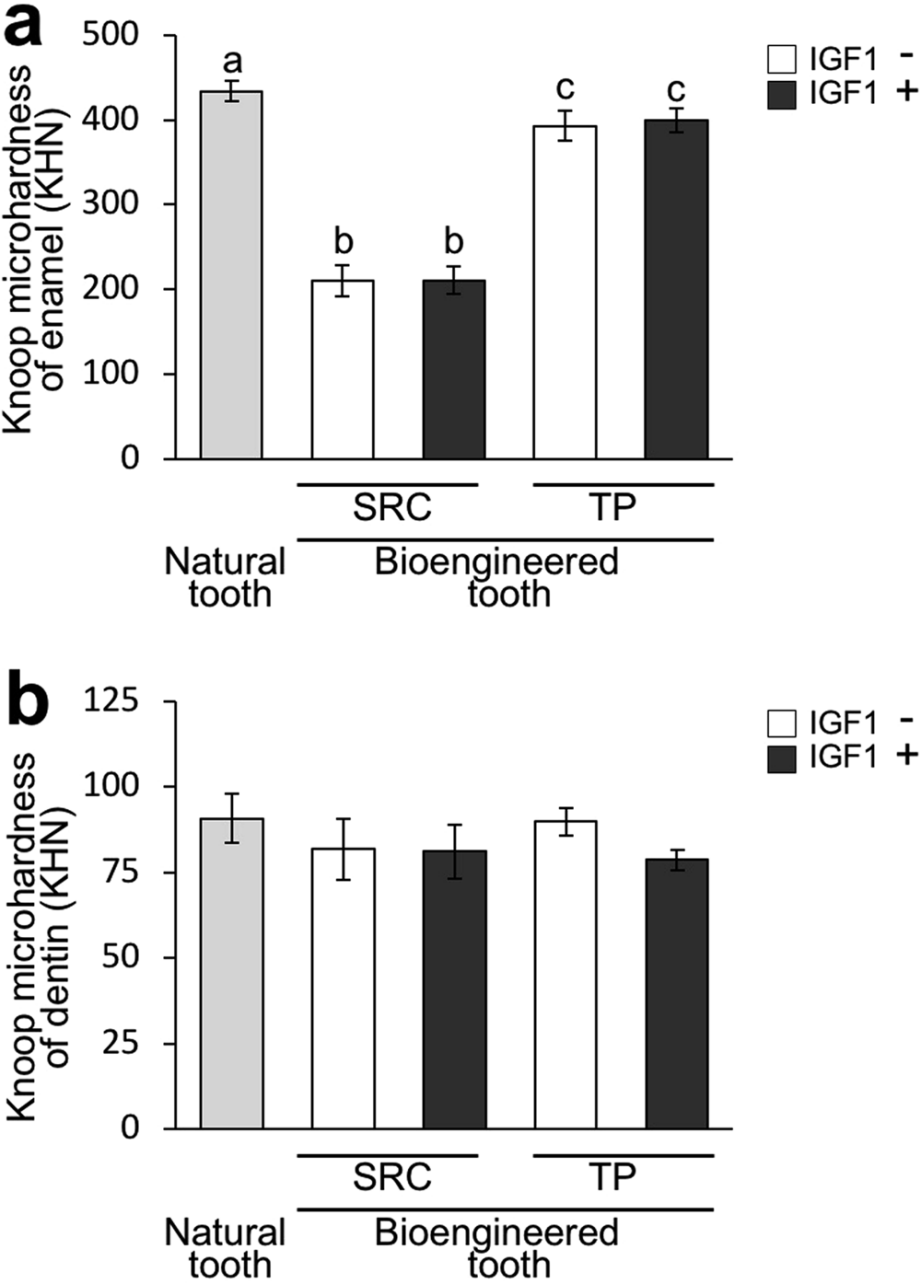
IGF1 does not affect the hardness of bioengineered teeth. The Knoop hardness values of the enamel (**a**) and dentin (**b**) of bioengineered teeth in the control and IGF1-treated groups at 30 days after transplantation into subrenal capsules (SRC), and at 90 days after transplantation into jawbones (TP), were assessed and compared with those of the upper first molars in 9-weeek-old-C57BL/J mice (NT, natural tooth). Error bars indicate the standard deviation (N = 5−7). The different letters (a, b, and c) indicate significant differences between groups. p < 0.05 (one-way analysis of variance followed by the Tukey-Kramer test).

To analyse the effect of IGF1 on the hardness of the bioengineered teeth, we measured Knoop hardness. The Knoop hardness number (KHN) value of the enamel of natural teeth was 433.7 ± 131.1 (N = 6) (Fig. 5a). The KHN values of the enamel of bioengineered teeth developed by renal subcapsular transplantation showed no significant difference between the control group (201.6 ± 19.9 KHN, N = 7) and the group treated with 4 µg/mL IGF1 (210.9 ± 17.9 KHN, N = 5); notably, both were significantly lower than the value of natural tooth enamel (Fig. 5a). The KHN values of the enamel of bioengineered teeth developed by transplantation into jawbones showed no significant difference between the control group (393.1 ± 20.1 KHN, N = 5) and the group treated with 4 µg/mL IGF1 (404.6 ± 19.2 KHN, N = 5); these values were similar to the value of natural tooth enamel (Fig. 5a). The KHN values of the dentin of the bioengineered teeth developed in subrenal capsules were 81.7 ± 9.5 (N = 7) in the control group and 81.1 ± 8.5 (N = 5) in the group treated with 4 µg/mL IGF1 (Fig. 5b). The KHN values of the dentin of the bioengineered teeth developed in jawbones were 89.7 ± 4.6 (N = 5) in the control group and 79.9 ± 4.2 (N = 5) in the group treated with 4 µg/mL IGF1 (Fig. 5b). These dentin KHN values were comparable to those of natural teeth (90.7 ± 7.1 KHN, N = 6) (Fig. 5b).

### IGF1 increases the number of *Fgf4*-expressing enamel knots during the development of bioengineered tooth germs

**Figure 6:**
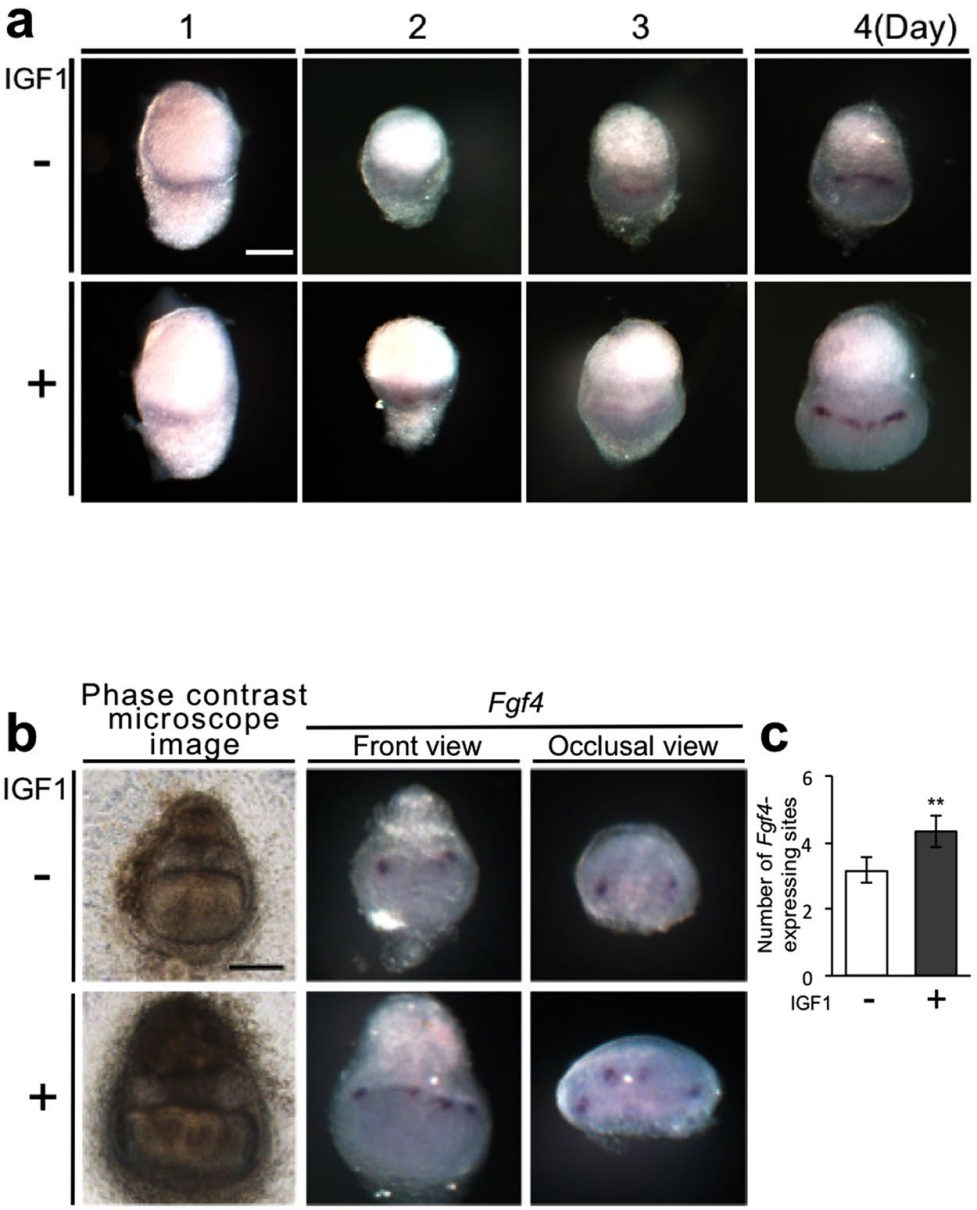
IGF1 increases the number of *Fgf4*-expressing sites during the development of bioengineered tooth germs. (**a**) The expression of *Fgf4* in bioengineered tooth germs was examined by whole-mount *in situ* hybridization at days 1, 2, 3, and 4 of organ culture in the control and IGF1-treated groups. Scale bar, 200 µm. (**b**) The expression of *Fgf4* in bioengineered tooth germs at day 5 of organ culture in the control and IGF1-treated groups was examined by whole-mount *in situ* hybridization. Scale bar, 200 µm. (**c**) The number of *Fgf4*-expressing sites was counted. Error bars indicate the standard deviation (N = 6). **p < 0.01 (versus control group; Student’s *t*-test).

The formation of enamel knots, a cluster of non-dividing epithelial cells, is a critical biological process for the patterning of tooth cusps^24,25^. To analyse the effect of IGF1 on enamel knot formation, we measured the expression of the enamel knot marker, *Fgf4*, during the development of the bioengineered tooth germs^24,25^. *Fgf4* expression was not detectable at day 1 (Fig. 6a). Its expression was observed in both groups at day 2; notably, the number of *Fgf4*-expressing sites increased during the development of the bioengineered tooth germs (Fig. 6a). The number of *Fgf4*-expressing sites at day 5 in the group treated with 4 µg/mL IGF1 (4.3 ± 0.5, N = 6) was significantly larger than that of the control group (3.2 ± 0.4, N = 6) (Fig. 6b,c).

### IGF1 directly upregulates the proliferation of both mesenchymal and epithelial cells derived from tooth germs

**Figure 7:**
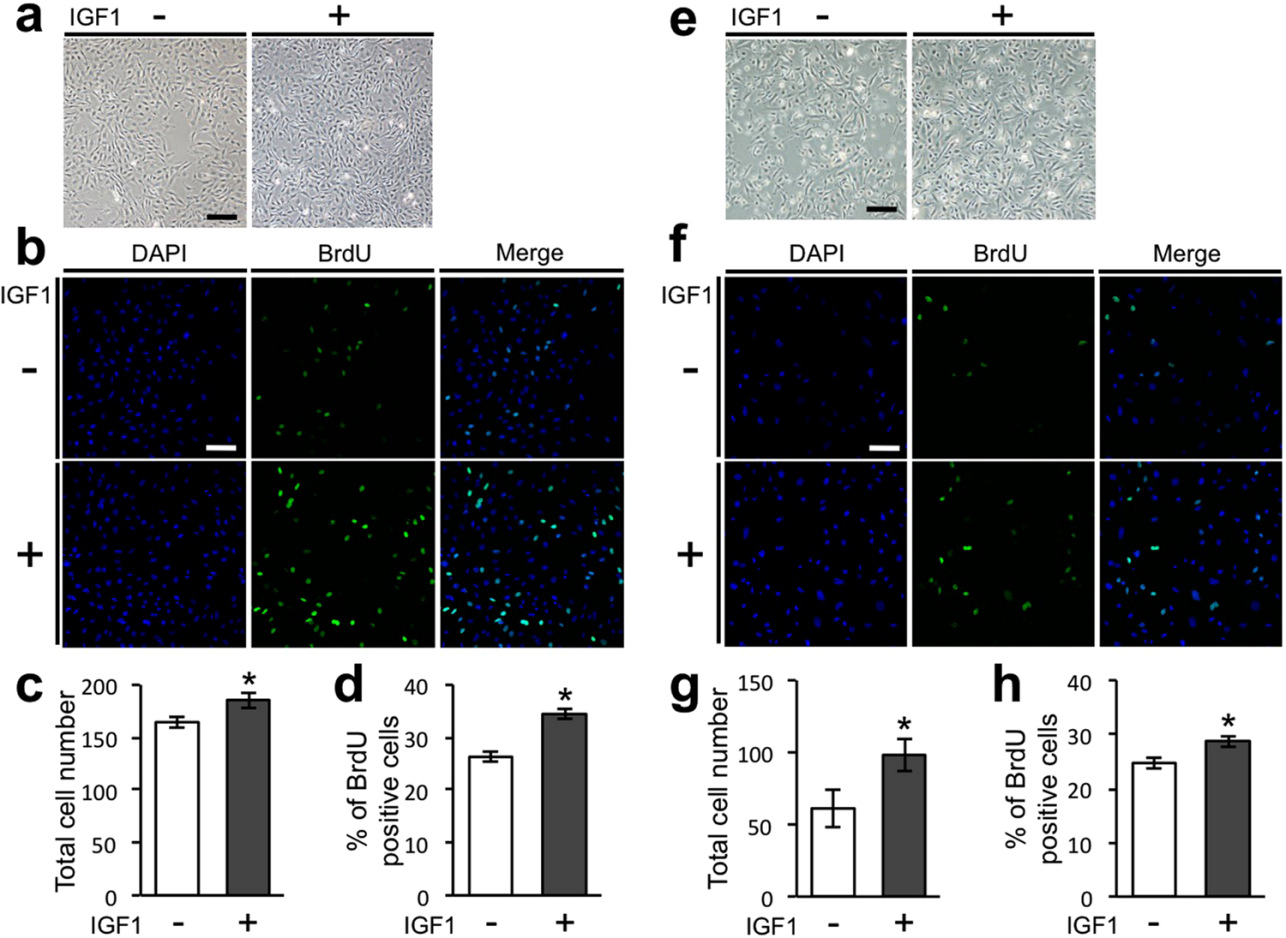
IGF1 directly upregulates the proliferation of mesenchymal and epithelial cells derived from tooth germs. (**a**) Phase-contrast images of mesenchymal cells isolated from mandibular molar tooth germs of ED14.5 mice at day 3 of culture. Scale bar, 200 µm. (**b**) BrdU-positive dental mesenchymal cells (green) were analysed by immunofluorescence. Nuclei were stained with DAPI (blue). Scale bar, 100 µm. Total numbers of dental mesenchymal cells (**c**) and percentages of BrdU-positive cells (**d**) were analysed on the immunofluorescent images. Error bars indicate the standard deviation (N = 3). *p < 0.05 (versus control group; Student’s *t*-test). (**e**) Phase-contrast images of epithelial cells isolated from mandibular molar tooth germs of ED14.5 mice at day 2 of culture. Scale bar, 200 µm. (**f**) BrdU-positive dental epithelial cells (green) were analysed by immunofluorescence. Nuclei were stained with DAPI (blue). Scale bar, 100 µm. The total numbers of dental epithelial cells (**g**) and percentages of BrdU-positive cells (**h**) were analysed on the immunofluorescent images. Error bars indicate the standard deviation (N = 3). *p < 0.05 (versus control group; Student’s *t*-test).

To investigate the mechanisms of the IGF1-mediated enhancement in the size of bioengineered teeth, we analysed the effect of IGF1 on the proliferation of dental mesenchymal and epithelial cells isolated from ED14.5 mouse tooth germs. The total cell number and percentage of bromodeoxyuridine (BrdU)-positive mesenchymal cells were significantly higher in the IGF1-treated group than in the control group (Fig. 7a−d). A significant increase in the total cell number and percentage of BrdU-positive epithelial cells was also observed in the IGF1-treated group (Fig. 7e–h).

### IGF1 stimulates the differentiation of mesenchymal and epithelial cells derived from tooth germs

**Figure 8:**
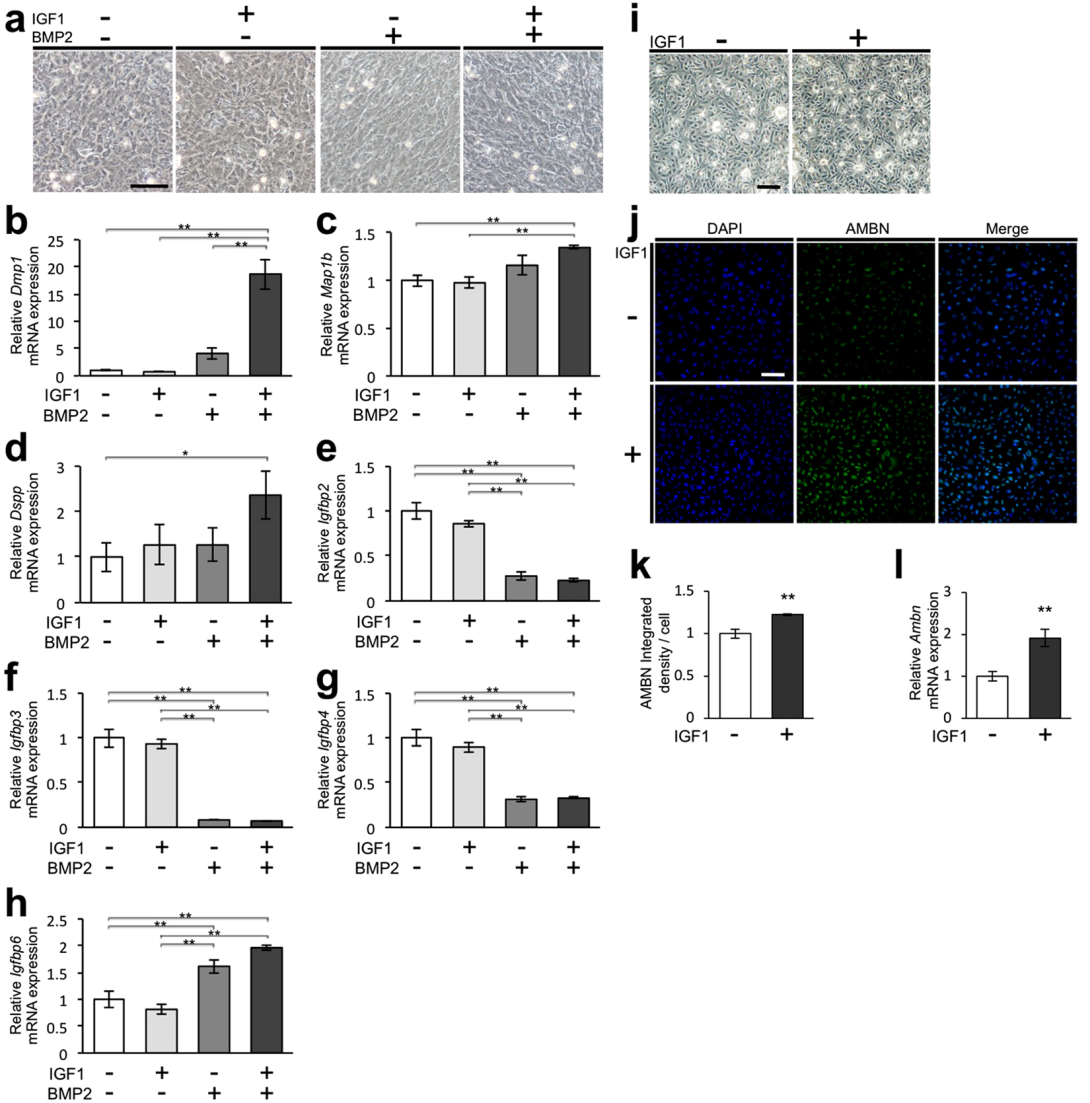
IGF1 stimulates the differentiation of mesenchymal and epithelial cells derived from tooth germs. (**a**) Phase-contrast images of mesenchymal cells isolated from the mandibular molar tooth germ of ED14.5 mice at day 10 of culture in the control, IGF1-treated, BMP2-treated, and IGF1 plus BMP2-treated groups. Scale bar, 100 µm. mRNA expression levels of *Dmp1* (**b**), *Map1b* (**c**), *Dspp* (**d**), *Igfbp2* (**e**), *Igfbp3* (**f**), *Igfbp4* (**g**) and *Igfbp6* (**h**) were quantitatively analysed by real-time PCR. Error bars indicate the standard deviation (N = 3). *p < 0.05, **p < 0.01 (one-way analysis of variance followed by the Tukey-Kramer test). (**i**) Phase-contrast images of epithelial cells isolated from the mandibular molar tooth germs of ED14.5 mice at day 4 of culture in the control and IGF1-treated groups. Scale bar, 200 µm. (**j**) Expression of AMBN (green) in the dental epithelial cells was analysed by immunofluorescence. Nuclei were stained with DAPI (blue). Scale bar, 100 µm. (**k**) The immunofluorescent signal of AMBN was quantitatively analysed. Data are expressed as integrated density/cell. Error bars indicate the standard deviation (N = 3). **p < 0.01 (versus control group; Student’s *t*-test). (**l**) The *Ambn* mRNA expression was quantitatively analysed by real-time PCR. Error bars indicate the standard deviation (N = 3). **p < 0.01 (versus control group; Student’s *t*-test).

Previous reports indicate that the interaction of IGF1 and bone morphogenetic protein 2 (BMP2) signalling contributes to the development of various tissues, including tooth, bone, and cartilage^26–28^. Both *Igf1* and *Igf1r* were expressed in dental epithelial and mesenchymal tissues in the ED14.5 tooth germs, while *Bmp2* was expressed solely in dental epithelial tissue (Supplementary Figs S6 and S7). We then examined the effect of IGF1 on the odontoblast differentiation of dental mesenchymal cells, focusing on its potential interaction with BMP2. Phase-contrast micrographs revealed a polygonal cell shape in the control and IGF1-treated groups at day 10 of culture (Fig. 8a). In contrast, mesenchymal cells in the BMP2-treated and BMP2 plus IGF1-treated groups were spindle-shaped (Fig. 8a). *Dentin sialophosphoprotein* (*Dspp*), *Dmp1*, and *microtubule-associated protein 1b* (*Map1b*) are known as odontoblast markers^21,29^. Expression of *Dmp1*, *Map1b*, and *Dspp* was clearly induced by the simultaneous administration of IGF1 and BMP2, whereas treatment with IGF1 or BMP2 alone did not induce expression of these markers (Fig. 8b–d). Next, we examined the effect of IGF1 and BMP2 on the expression of insulin-like growth factor-binding proteins (IGFBPs), which regulate IGF1 bioactivity^30^. The expression levels of *Igfbp2*, 3, and 4 were significantly reduced, while the expression of *Igfbp6* was significantly enhanced by BMP2 treatment, as well as by simultaneous administration of IGF1 and BMP2 (Fig. 8e–h).

We examined the effect of IGF1 on the differentiation of dental epithelial cells. The epithelial cells reached confluence and showed polygonal shape at day 4 of culture (Fig. 8i). IGF1 treatment did not visibly alter the shape of the epithelial cells (Fig. 8i). *Ambn* is considered a marker of ameloblast differentiation^19,20^. Analysis of *Ambn* expression demonstrated significantly greater expression of *Ambn* in the IGF1-treated group than in the control group (Fig. 8j–l).

## Discussion

In the present study, we analysed the effect of IGF1 on the morphology of bioengineered teeth. We demonstrated that IGF1 treatment significantly increased the size of bioengineered tooth germs while maintaining normal dental histological architecture. IGF1 binds to IGF1R with high affinity and activates its signalling pathway^31,32^. IGF1R expression was observed in ameloblasts, odontoblasts, and dental papilla cells during the development of bioengineered tooth germs; these IGF1R expression patterns were similar to those of normal tooth germs^33–35^. Our findings suggest that IGF1 increases the size of bioengineered tooth germs by acting on both epithelial and mesenchymal cells. Moreover, the IGF1-treated bioengineered teeth, which were developed from bioengineered tooth germs in subrenal capsules and jawbones, showed an increase not only in size but also in cusp number. These findings indicated for the first time that IGF1 is a competent agent for increasing the size and cusp number of bioengineered teeth. Importantly, IGF1 treatment did not interfere with the formation of PDL surrounding tooth roots and the acquisition of significant hardness of enamel and dentin. PDL has essential roles in tooth function, such as perception, adaptation to mechanical load, and homeostasis of teeth and periodontal tissues^36^. Sufficient tooth hardness is required to exert masticatory potential^37^. Therefore, the IGF1-treated bioengineered teeth could exert normal physiological function as related to PDL and tooth hardness.

The biological effects of pharmaceutical agents are dependent on their concentration. We demonstrated that IGF1 increased the size of bioengineered teeth in a dose-dependent manner, representing maximum effects at 4 μg/mL. The physiological concentration of IGF1 in the serum of 4–8-week-old mice is approximately 0.4–1 μg/mL^8,38^. Therefore, the concentration of IGF1 used in the present study is higher than the physiological level. However, the histological structure and the expression patterns of IGF1R and enamel and dentin matrix proteins in the bioengineered tooth germs treated with 4 μg/mL IGF1 showed no abnormal changes when compared with the control group. In addition, pathological findings such as cell necrosis and tissue degeneration were not observed. Therefore, the concentration of IGF1 used in the present study had no adverse effect on the bioengineered tooth germs.

We transplanted the bioengineered tooth germs, which had been cultured with IGF1 for 7 days, into mouse subrenal capsules and jawbones. Although exogenous IGF1 was not administered after the transplantation, the generated bioengineered teeth showed increased size and cusp number. Kim *et al*.^39^ cultured tooth germs extracted from ED13.5 mice with the administration of a caspase inhibitor for 2 days, and subsequently transplanted them into mouse subrenal capsules. As a result, teeth with shorter crown heights and wider mesiodistal diameters were generated despite no further administration of the inhibitor to the mice. These findings indicate that the *ex vivo* effects of agents on the morphology of bioengineered tooth germs are maintained during their development into bioengineered teeth in the living body. This concept is significant for the future clinical application of IGF1 to regulate the morphology of bioengineered teeth.

During tooth germ development, epithelial cells form the enamel knot at the tip of the cap-shaped tooth germ (cap stage)^40^. During the progression of tooth development, the number of enamel knots increases and the location of future cusps is determined^25^. BMP2 expression in dental epithelium induces the formation of enamel knots^41^. BMP4 expression in dental mesenchyme also contributes to enamel knot formation via upregulation of p21 and Msh homeobox 1 (Msx1)^41^. However, the molecular mechanism of enamel knot formation is not fully understood. In the present study, IGF1 increased the number of *Fgf4*-expressing sites in bioengineered tooth germs, indicating that IGF1 directly and/or indirectly contributes to the production of enamel knots. FGF4 expression in enamel knots upregulates the proliferation of peripheral dental epithelial and mesenchymal cells during tooth morphogenesis^24^. Kratochwil *et al*.^42^ reported that FGF4 expression in enamel knots induces the expression of SHH in dental epithelium, which is an essential factor for tooth morphogenesis, via upregulation of FGF3 in dental mesenchyme. Our data suggest that the IGF1-induced FGF4 in enamel knots could activate proliferation and downstream signalling, subsequently increasing the size and cusp number of bioengineered teeth. The IGF1-dependent increase in the number of enamel knots could subsequently increase the cusp number in bioengineered teeth. On the other hand, generation of more space between enamel knots during growth of tooth germs would allow the formation of new enamel knots, regulated by activators and inhibitors of enamel knot formation^43^. Thus, it is possible that the IGF-induced increase in the size of bioengineered tooth germs indirectly increases the total number of enamel knots via activator-inhibitor loops. We need further study to elucidate regulatory mechanisms of the IGF-induced increase in the size and cusp number of bioengineered teeth.

Regarding the role of IGF1 in the proliferation of tooth germ cells, Young *et al*.^15^ showed an inducible effect of IGF1 on mitotic activity in the dental epithelial cells of ED16 mouse molar tooth germs. Caton *et al*.^44^ reported that IGF1 induces proliferation of dental mesenchymal cells isolated from ED18 mouse molar tooth germs. However, the effect of IGF1 on the proliferation of mesenchymal and epithelial cells in stage of tooth germ development earlier than ED16 has not been clarified. In the present study, we revealed for the first time, that IGF1 directly enhanced the proliferation of both dental mesenchymal and epithelial cells isolated from the tooth germs of ED14.5 mice *in vitro*. In the molar tooth germ of ED14 mice, epithelial tissue invaginates around the mesenchymal tissue, reaching the cap stage^45^. Subsequently, the size of tooth germs increases as the dental epithelial tissue further invaginates and elongates into mesenchymal tissue by active cell proliferation; the tooth germ then forms a bell-shaped crown at ED16 (bell stage)^45^. Thus, our data suggest that IGF1 contributes to increased tooth germ size by stimulating the proliferation of dental mesenchymal and epithelial cells between the cap and bell stages. The direct effect of IGF1 on proliferation may provide a mechanism to increase the size of bioengineered teeth.

Dental mesenchymal cells begin to differentiate into odontoblasts by signallings from the enamel knot in the bell stage^46^; dental epithelial cells begin to differentiate into ameloblasts following odontoblast differentiation^46^. During the late bell stage, differentiated odontoblasts and ameloblasts form dentin and enamel, which are dental hard tissues, and teeth develop with increasing size^38^. Thus, we analysed the effect of IGF1 on odontoblast and ameloblast differentiation of dental mesenchymal and epithelial cells isolated from ED14.5 mouse molar tooth germs, in order to further elucidate the mechanism for the increased size of bioengineered teeth.

In the present study, we showed that expression of odontoblast markers *Dmp1*, *Dspp*, and *Map1b* was not induced by IGF1 in dental mesenchymal cells. Expression of these markers was also not induced by BMP2. In contrast, simultaneous administration of IGF1 and BMP2 resulted in significant induction of these marker genes. Bègue-Kirn *et al*.^47^ also showed that the combination of IGF1 and BMP2 promotes odontoblast differentiation by histological analysis of organ-cultured dental papilla tissue isolated from ED17 mouse tooth germs; IGF1 or BMP2 alone does not promote this differentiation. These findings suggest that BMP2 is essential for IGF1-induced differentiation of dental mesenchymal cells into odontoblasts.

We then analysed expression of IGFBPs, which regulate the biological action of IGF1, to elucidate the mechanism by which simultaneous administration of IGF1 and BMP2 induces differentiation of dental mesenchymal cells into odontoblasts. We found decreased expression of *Igfbp2*, 3, and 4; notably, expression of *Igfbp6* was increased by BMP2, with or without IGF1 administration. Takahashi *et al*.^28^ showed that BMP2 decreased the expression of IGFBP3 in neonatal bovine chondrocytes. These findings suggest that BMP2 is a modulator of IGFBP expression. IGFBP2, 3, and 4 inhibit the bioactivity of IGF1 by interfering with IGF1 binding to its receptor^30^. Thus, activation of IGF1 signalling due to reduced expression of IGFBP2, 3, and 4 by BMP2 constitutes the mechanism by which simultaneous administration of IGF1 and BMP2 induces differentiation of dental mesenchymal cells into odontoblasts. Additionally, IGFBP6 has high binding affinity for IGF2 and suppresses the activity of IGF2^30^. However, IGFBP6 has low binding affinity for IGF1 and its effect on IGF1 activity remains uncleared^30^. Moreover, although IGFBP6 suppresses the differentiation of oligodendrocyte precursor cells, and osteoblasts^48,49^, its role in the differentiation of dental cells has not been reported. Further studies are necessary to clarify the role of BMP2-induced IGFBP6 in odontoblast differentiation.

In the present study, we showed that the expression of the enamel matrix gene *Ambn* was enhanced upon IGF1 treatment of cultured dental epithelial cells. Takahashi *et al*.^50^ showed that IGF1 increased the expression of *Ambn* in organ-cultured ED15 mouse molar tooth germs. However, it is not clear whether IGF1 directly increases the expression of *Ambn* in dental epithelial cells, because tooth development progresses through various signalling crosstalk mechanisms between epithelial and mesenchymal tissues. The present study showed that the expression of *Ambn* was directly enhanced by IGF1 in dental epithelial cells. *Ambn* is expressed earlier than other enamel matrix genes, such as *Enam* and *Amel*, during tooth germ development^51^. Because *Ambn*-null mice exhibited severe enamel hypoplasia and reduced expression of *Amel*, *Ambn* may play an essential role in normal ameloblast differentiation^20^. Therefore, IGF1-induced *Ambn* would induce the ameloblasts differentiation during the development of bioengineered tooth germs.

IGF1 plays a role in increasing the size of various organs, such as the kidneys, liver, spleen, and bones, during postnatal growth of rats and mice^52,53^. Therefore, our findings contribute to a novel strategy for the morphNological control of bioengineered organs (not strictly limited to teeth) by the activation of IGF1 signalling.

## Methods

### Ethics statement

C57BL/6 mice were purchased from CLEA Japan (Tokyo, Japan). Animal experiments were performed in accordance with the Regulations for Animal Experiments and Related Activities at Tohoku University. All animal protocols were approved by the Institutional Animal Care and Use Committee of the Tohoku University Environmental and Safety Committee.

### Generation of bioengineered tooth germs from single cells

Bioengineered tooth germs were generated as described previously^2^. Molar tooth germs were dissected from the mandibles of ED14.5 C57BL/6 mice. To generate tissue recombinants, 5.0 × 10^4^ epithelial and mesenchymal cells each in 0.05 µL were injected into 30-µL collagen gel drops of Cellmatrix type I-A (Nitta Gelatin, Osaka, Japan) using a Hamilton syringe (Osaka Chemical, Osaka, Japan). Recombinant mouse IGF1 (R&D Systems, McKinley, Minneapolis, MN, USA) (0.5, 1, 2, 4, and 8 µg/mL) was added to the gel drops in the IGF1-treated group.

### Organ culture

Collagen gel drops with tissue recombinants were incubated for 10 min at 37 °C, transferred to a cell culture insert (0.4-µm pore diameter; Becton Dickinson, Franklin Lakes, NJ, USA) in 24-well cell culture plates (BD), and cultured with 250 µL/well Dulbecco’s modified eagle medium (DMEM; Sigma, St. Louis, MO, USA) supplemented with 10% fetal bovine serum (FBS; Hyclone, Logan, UT, USA), 100 units/mL of penicillin, and 100 µg/mL of streptomycin (Invitrogen, Carlsbad, CA, USA). On the next day, the culture medium was changed to DMEM containing 10% FBS, 100 units/mL of penicillin, 100 µg/mL of streptomycin, 2 mM L-glutamine (Sigma), and 100 µg/mL ascorbic acid (Sigma). The medium was then changed every other day. The tooth germs were dissected from mandibles of ED14.5 mice and then cultured in accordance with the same protocol as for tissue recombinants.

### Transplantation of bioengineered tooth germs into subrenal capsules

Bioengineered tooth germs isolated from collagen gel after 7-day-organ culture were placed into plastic ring-shaped devices with 2.5-mm internal diameter and 1.3-mm thickness. These were transplanted into subrenal capsules of 7-week-old C57BL/6 mice under general anaesthesia, as described previously^54^.

### Transplantation of tooth germs into jawbones

Transplantation of bioengineered tooth germs into jawbones was performed as described previously^3^. Upper first molars of 5-week-old C57BL/6 mice were extracted under general anaesthesia and maintained for 3 weeks to allow natural repair of the tooth cavity and oral epithelium. Then, the oral mucosa at the extraction sites was incised and bony holes (0.8 mm in diameter and 1.5 mm in depth, one hole per site) were made by a dental micro motor (PAL; Morita, Osaka, Japan). Bioengineered tooth germs were isolated from collagen gel after 7-day-organ culture, marked at the top of the dental epithelium with methylene blue to confirm the direction, and transplanted into the bony holes. The incised oral mucosa was sutured with 8–0 nylon (BEAR Medic, Chiba, Japan).

### Micro-computed tomography (Micro-CT)

Micro-CT images of bioengineered teeth developed in subrenal capsules and jawbones were obtained by R_mCT2 (Rigaku, Tokyo, Japan) or Latheta LCT-200 (Hitachi, Aloka Medical, Tokyo, Japan). The micro-CT images were reconstructed and analysed using i-VIEW-3DX (Morita) or TRI/3D-BON (Ratoc System Engineering, Tokyo, Japan).

### Measurement of Knoop hardness

Bioengineered teeth were extracted 30 and 90 days after transplantation into subrenal capsules and jawbones, respectively. Upper first molars of 9-week-old C57BL/6 mice were also extracted. These samples were immersed in 4% paraformaldehyde (PFA); then, flat surfaces of enamel and dentin were made. Knoop hardness was measured using a Miniload Hardness Tester (Mitutoyo, Tokyo, Japan) equipped with a Knoop diamond tip (Mitutoyo). Five indentations were made on each sample with a 10-g load for 10 sec.

### Histological analysis

Bioengineered and natural tooth germs, as well as jawbones with bioengineered teeth, were immersed in 4% PFA and decalcified in 4.5% EDTA at 4 °C, as described previously^2^. After decalcification, the samples were embedded in paraffin and cut into sections. The sections were deparaffinized and stained with haematoxylin and eosin (HE).

### Immunohistochemistry

To examine the expression of AMBN, deparaffinized sections were immersed in 0.1 M citrate buffer, boiled by microwave for 2 min, and incubated at room temperature for 30 min. The sections were washed with phosphate-buffered saline (PBS), treated with 3% H_2_O_2_ in methanol for 15 min at room temperature, and incubated with an anti-AMBN antibody (1:500, diluted in PBS; Santa Cruz Biotechnology, Dallas, TX, USA) at 4 °C overnight. The sections were incubated with Histofine Simplestain Max PO (Nichirei, Tokyo, Japan) for 30 min at room temperature. Signals were detected by reaction with 3,3′-diaminobenzidine tetrahydrochloride (DAB; Nichirei, Tokyo, Japan).

Deparaffinized sections were immersed in 0.1 M citrate buffer for 2 h at 37 °C to examine the expression of IGF1R and in 1.0 mg/mL hyaluronidase (Sigma) for 1 h at 37 °C to examine the expression of DMP1 and DSP. The sections were treated with 3% H_2_O_2_ for 5 min at room temperature and then incubated with anti-IGF1R (1:50; Merck Millipore, Darmstadt, Germany), anti-DMP1 (1:50; Merck Millipore), or anti-DSP (1:250; Merck Millipore) antibodies for 30 min at room temperature using the VECTOR M.O.M Immunodetection Kit (Vector Laboratories, Burlingame, CA, USA), in accordance with the manufacturer’s protocol. The sections were incubated with VECTASTAIN Elite ABC Reagent (Vector Laboratories) and signals were detected by reaction with DAB.

To analyse the expression of NF and VWF, deparaffinized sections were treated with 0.15% Triton-X100 in PBS for 30 min at room temperature. After sections were blocked with 1% goat serum (Vector Laboratories) in PBS for 1 h at room temperature, they were incubated with anti-NF (1:200; Chemicon, Temecula, CA, USA) or anti-VWF (1:1000; Dako, Glostrup, Denmark) antibodies for 2 h at room temperature. After sections were washed with 0.1% Tween 20 in PBS, they were treated with goat anti-rabbit IgG Alexa 488 (1:1000; Invitrogen) for 1 h at room temperature. Nuclei were stained with 4’,6-diamidino-2-phenylindole (DAPI; 1:1000; KPL, Gaithersburg, MD, USA). Samples were visualized using a confocal laser scanning microscope system (C2si; Nikon Instech, Tokyo, Japan).

### Immunocytochemistry

Epithelial cells isolated from the mandibular first molar tooth germs of ED14.5 C57BL/6 mice were seeded at a density of 1.5 × 10^5^ cells/cm^2^ on glass-bottomed dishes coated with 20 µg/mL fibronectin (Sigma) and 100 µg/mL type IV collagen (Nitta Gelatin) and cultured in CnT-Prime medium (CELLnTEC, Bern, Switzerland). On the next day, 500 ng/mL IGF1 was added into the medium. After 4-day culture, cells were fixed using 4% PFA for 15 min and treated with 0.1% Triton-X100 in PBS for 15 min at room temperature. After cells were blocked with 3% bovine serum albumin (Sigma) in PBS for 1 h at room temperature, they were incubated with an anti-AMBN antibody (1:100; Santa Cruz Biotechnology Inc.) overnight at 4 °C. Cells were treated with goat anti-rabbit IgG Alexa 488 (1:1000; Invitrogen) for 1 h at room temperature. Nuclei were stained with DAPI (1:1000; KPL). Signals were visualized using C2si (Nikon Instech). Integrated density measurements of five microscopic fields in each sample were performed using ImageJ software (https://imagej.nih.gov/ij/).

### Probe preparation and whole-mount *in situ* hybridization

Digoxigenin-labelled RNA probes were prepared with the MAXIscript Kit (Thermo Fisher Scientific, Waltham, MA, USA), in accordance with the manufacturer’s instructions. The DNA fragment of *Fgf4* (NM_010202, located between 117 and 731) was amplified by polymerase chain reaction (PCR), using primers shown in Supplementary Table S1. The fragment was subcloned into the pGEM-T vector (Promega, Madison, WI, USA) and used to generate sense and antisense probes. Bioengineered tooth germs after 5-day organ culture were fixed in 4% PFA overnight at 4 °C, treated with 6% H_2_O_2_ and 0.1% Tween 20 in PBS at room temperature, and incubated with 10 µg/mL proteinase K (Roche, Mannheim, Germany) for 3 min at 30 °C. After post-fixation and prehybridization, samples were hybridized overnight at 70 °C with digoxigenin-labelled RNA probes. Samples were blocked with 10% normal sheep serum (Sigma) and then incubated with anti-digoxigenin antibody conjugated with alkaline phosphatase (Roche), overnight at 4 °C. Nitro blue tetrazolium and 5-bromo-4-chloro-3-indolyl phosphate (Roche) were used for signal detection.

### BrdU assay

Mesenchymal and epithelial cells were isolated from the mandibular molar tooth germs of E14.5 C57BL/6 mice. Mesenchymal cells were seeded on glass-bottomed dishes at a density of 2.0 × 10^4^ cells/cm^2^ and cultured in DMEM supplemented with 10% FBS, 100 units/mL of penicillin, and 100 µg/mL of streptomycin. On the next day, IGF1 (500 ng/mL) was added into the medium. Epithelial cells were seeded at a density of 7.5 × 10^4^ cells/cm^2^ on glass-bottomed dishes coated with 20 µg/mL fibronectin (Sigma) and 100 µg/mL type IV collagen (Nitta Gelatin); the cells were cultured in CnT-Prime medium (CELLnTEC). After 3-day culture of mesenchymal cells and 2-day culture of epithelial cells, cells were incubated with DMEM supplemented with 1% BrdU Labeling Reagent (Invitrogen) for 4 h at 37 °C, then fixed with 70% ethanol for 15 min at 4 °C. The cells were treated with 0.1% Triton-X100 in PBS for 20 min at room temperature, then with Denaturing Solution (BrdU Staining Kit, Invitrogen) for 30 min at room temperature. Cells were treated with 0.1% Triton-X100 and 5% normal goat serum in PBS for 1 h at room temperature and incubated with Alexa Fluor 488-conjugate BrdU antibody (1:100; Thermo Fisher Scientific) overnight at room temperature. Nuclei were stained with DAPI (1:1000; KPL). Samples were visualized using C2si (Nikon Instech). Counts of total and BrdU-positive cell numbers were performed using five microscopic fields in each sample.

### PCR

Total RNA was isolated from whole mandibular first molar tooth germ, dental mesenchymal and epithelial tissues, and mesenchymal and epithelial cells isolated from tooth germs in E14.5 mice, using the RNeasy Kit (Qiagen, Valencia, CA, USA). cDNA was synthesized from 0.4 µg total RNA using the PrimeScript RT Reagent Kit (Takara Bio, Shiga, Japan). Conventional reverse transcription-PCR (RT-PCR) was performed by Veriti™ 96-Well Thermal Cycler (Applied Biosystems, Foster City, CA, USA). The reaction volume was 50 µL, which contained 2 µL of cDNA, 0.4 µM of sense and antisense primer, and 25 µL of EmeraldAmp^®^ PCR Master Mix (Takara Bio). The reactions consisted of gene-specific numbers of cycles (*Pitx2*: 28 cycles, *Shh*: 32 cycles, *Fgf4*: 28 cycles, *Fgf10*: 35 cycles, *Igf1*: 32 cycles, *Igf1r*: 30 cycles, *Bmp2*: 31 cycles, and *glyceraldehyde-3-phosphate dehydrogenase* (*Gapdh*): 30 cycles) of 10 sec at 98 °C for thermal denaturation and 60 sec at 60 °C for annealing and elongation. The cDNA obtained by the PCR reaction was separated by electrophoresis using a 2% agarose gel containing ethidium bromide. Real-time PCR was performed by using the Thermal Cycler Dice Real-Time system (Takara Bio). The reaction volume was 25 µL, which contained 2 µL of cDNA, 0.4 µM of sense and antisense primer, and 12.5 µL of SYBR Premix Ex Taq II (Takara Bio). The reactions consisted of 40 cycles of 5 sec at 95 °C and 30 sec at 60 °C. The mRNA expression of each sample was normalized to that of *Gapdh* and analysed by the ΔΔCT method. Primers used for RT-PCR and real-time PCR are listed in Supplementary Table S2.

### Statistical analysis

Data were statistically analysed by Student’s t-test to compare differences between two groups. For more than two groups, we used an analysis of variance (ANOVA) followed by the Tukey-Kramer test. P values < 0.05 was considered statistically significant.

## Supplementary information

Supplementary Information

## References

[1] Zhang YD, Chen Z, Song YQ, Liu C, Chen YP (2005). Making a tooth: growth factors, transcription factors, and stem cells. Cell Res..

[2] Nakao K (2007). The development of a bioengineered organ germ method. Nat. Methods.

[3] Ikeda E (2009). Fully functional bioengineered tooth replacement as an organ replacement therapy. Proc. Natl. Acad. Sci. USA.

[4] Lucas, P. W. *Dental functional morphology*. How *teeth work. (ed. Lucas, P. W.)* 166–167 (Cambridge University, 2004).

[5] Berthaume, M. A., Dumont, E. R., Godfrey, L. R. & Grosse, I. R. The effects of relative food item size on optimal tooth cusp sharpness during brittle food item processing. *J. R. Soc. Interface* **11**, 10.1098/rsif.2014.0965 (2014). 10.1098/rsif.2014.0965 PMC4223924 25320068

[6] Thesleff I (2003). Epithelial-mesenchymal signalling regulating tooth morphogenesis. J. Cell Sci..

[7] Powell-Braxton L (1993). IGF-I is required for normal embryonic growth in mice. Genes Dev..

[8] Liu JL, LeRoith D (1999). Insulin-Like Growth Factor I Is Essential for Postnatal Growth in Response to Growth Hormone. Endocrinology.

[9] AI-Kharobi H, EI-Gendy R, Devine DA, Beattie J (2014). The role of the insulin-like growth factor (IGF) axis in osteogenic and odontogenic differentiation. Cell. Mol. Life Sci..

[10] Fant ME, Weisoly D (2001). Insulin and insulin-like growth factors in human development: implications for the perinatal period. Semin. Perinatol..

[11] Fukunaga T (2008). Dental and craniofacial characteristics in a patient with leprechaunism treated with insulin-like growth factor-I. Angle Orthod..

[12] Sarnat H, Kaplan I, Pertzelan A, Laron Z (1988). Comparison of Dental Findings in Patients With Isolated Growth-Hormone Deficiency Treated With Human Growth-Hormone (Hgh) and in Untreated Patients With Laron-Type Dwarfism. Oral Surg. Oral Med. Oral Pathol. Oral Radiol. Endod..

[13] Joseph BK (1993). Expression and regulation of insulin-like growth factor-I in the rat incisor. Growth factors.

[14] Joseph BK, Savage NW, Young WG, Waters MJ (1994). Prenatal expression of growth hormone receptor/binding protein and insulin-like growth factor-I (IGF-I) in the enamel organ. Role for growth hormone and IGF-I in cellular differentiation during early tooth formation?. Anat. Embryol..

[15] Young WG (1995). Comparison of the effects of growth hormone, insulin-like growth factor-I and fetal calf serum on mouse molar odontogenesis *in vitro*. Arch. Oral Biol..

[16] Catón J, Bringas P, Zeichner-David M (2005). IGFs increase enamel formation by inducing expression of enamel mineralizing specific genes. Arch. Oral Biol..

[17] Hu B (2006). Tissue engineering of tooth crown, root, and periodontium. Tissue Eng..

[18] Ishida K (2011). The regulation of tooth morphogenesis is associated with epithelial cell proliferation and the expression of Sonic hedgehog through epithelial-mesenchymal interactions. Biochem. Biophys. Res. Commun..

[19] Simmons D, Gu TT, Krebsbach PH, Yamada Y, MacDougall M (1998). Identification and characterization of a cDNA for mouse ameloblastin. Connect. Tissue Res..

[20] Fukumoto S (2004). Ameloblastin is a cell adhesion molecule required for maintaining the differentiation state of ameloblasts. J. Cell Biol..

[21] D’Souza RN (1997). Gene expression patterns of murine dentin matrix protein 1 (Dmp1) and dentin sialophosphoprotein (DSPP) suggest distinct developmental functions *in vivo*. J. Bone Miner. Res..

[22] Cai J (2007). Patterning the size and number of tooth and its cusps. Dev. Biol..

[23] Takimoto A (2015). Scleraxis and osterix antagonistically regulate tensile force-responsive remodeling of the periodontal ligament and alveolar bone. Development.

[24] Jernvall J, Kettunen P, Karavanova I, Martin LB, Thesleff I (1994). Evidence for the role of the enamel knot as a control center in mammalian tooth cusp formation: Non-dividing cells express growth stimulating Fgf-4 gene. Int. J. Dev. Biol..

[25] Cho SW (2007). The primary enamel knot determines the position of the first buccal cusp in developing mice molars. Differentiation.

[26] Li H (1998). Growth hormone and insulin-like growth factor I induce bone morphogenetic proteins 2 and 4: a mediator role in bone and tooth formation?. Endocrinology.

[27] Celil AB, Campbell PG (2005). BMP-2 and insulin-like growth factor-I mediate Osterix (Osx) expression in human mesenchymal stem cells via the MAPK and protein kinase D signaling pathways. J. Biol. Chem..

[28] Takahashi T, Morris EA, Trippel SB (2007). Bone morphogenetic protein-2 and -9 regulate the interaction of insulin-like growth factor-I with growth plate chondrocytes. Int. J. Mol. Med..

[29] Maurin JC (2009). Microtubule-associated Protein 1b, a Neuronal Marker Involved in Odontoblast Differentiation. J. Endod..

[30] Collett-solberg PF, Cohen P (2000). Genetics, Chemistry, and Function of the IGF/IGFBP System. Endocrine.

[31] Rother KI, Accili D (2000). Role of insulin receptors and IGF receptors in growth and development. Pediatr. Nephrol..

[32] Nakae J, Kido Y, Accili D (2001). Distinct and Overlapping Functions of Insulin and IGF-I Receptors. Endocr. Rev..

[33] Joseph BK, Savage NW, Young WG, Waters MJ (1994). Insulin-like growth factor-I receptor in the cell biology of the ameloblast: an immunohistochemical study on the rat incisor. Epithelial Cell Biol..

[34] Magnucki G (2013). Expression of the IGF-1, IGFBP-3 and IGF-1 receptors in dental pulp stem cells and impacted third molars. J. Oral Sci..

[35] Kero D (2016). Involvement of IGF-2, IGF-1R, IGF-2R and PTEN in development of human tooth germ - an immunohistochemical study. Organogenesis.

[36] McCulloch CA, Lekic P, McKee MD (2000). Role of physical forces in regulating the form and function of the periodontal ligament. Periodontol. 2000.

[37] Zaslansky P, Friesem AA, Weiner S (2006). Structure and mechanical properties of the soft zone separating bulk dentin and enamel in crowns of human teeth: Insight into tooth function. J. Struct. Biol..

[38] Riquelme R (2010). A comparative study of age-related hearing loss in wild type and insulin-like growth factor I deficient mice. Front. Neuroanat..

[39] Kim JY (2006). Inhibition of apoptosis in early tooth development alters tooth shape and size. J. Dent. Res..

[40] Antonio, N. *Ten Cate’s Oral Histology: Development, Structure, and Function*. (ed. Antonio, N.) 80 (Mosby, 2013).

[41] Jernvall J, Åberg T, Kettunen P, Keränen S, Thesleff I (1998). The life history of an embryonic signaling center: BMP-4 induces p21 and is associated with apoptosis in the mouse tooth enamel knot. Development.

[42] Kratochwil K, Galceran J, Tontsch S, Roth W, Grosschedl R (2002). FGF4, a direct target of LEF1 and Wnt signaling, can rescue the arrest of tooth organogenesis in Lef1(−/−) mice. Genes Dev..

[43] Jernvall J, Jung HS (2000). Genotype, phenotype, and developmental biology of molar tooth characters. Am. J. Phys. Anthropol..

[44] Catón J, Bringas P, Zeichner-David M (2007). Establishment and characterization of an immortomouse-derived odontoblast-like cell line to evaluate the effect of insulin-like growth factors on odontoblast differentiation. J. Cell. Biochem..

[45] Morita, R. *et al*. Coordination of cellular dynamics contributes to tooth epithelium deformations. *PLoS One* **11**, 10.1371/journal.pone.0161336. (2016). 10.1371/journal.pone.0161336 PMC5010284 27588418

[46] Thesleff I, Keränen S, Jernvall J (2001). Enamel knots as signaling centers linking tooth morphogenesis and odontoblast differentiation. Adv. Dent. Res..

[47] Bègue-Kirn C (1994). Comparative analysis of TGF beta s, BMPs, IGF1, msxs, fibronectin, osteonectin and bone sialoprotein gene expression during normal and *in vitro*-induced odontoblast differentiation. Int. J. Dev. Biol..

[48] Kühl NM, Hoekstra D, De Vries H, De Keyser J (2003). Insulin-Like Growth Factor-Binding Protein 6 Inhibits Survival and Differentiation of Rat Oligodendrocyte Precursor Cells. Glia.

[49] Strohbach C (2008). Potential Involvement of the Interaction Between Insulin-Like Growth Factor Binding Protein (IGFBP) -6 and LIM Mineralization Protein (LMP) -1 in Regulating Osteoblast Differentiation. J. Cell. Biochem..

[50] Takahashi K (1998). Induction of amelogenin and ameloblastin by insulin and insulin-like growth factors (IGF-I and IGF-II) during embryonic mouse tooth development *in vitro*. Connect. Tissue Res..

[51] Gene expression in tooth. *Developmental Biology Programme of the University of Helsinki* Available at, http://bite-it.helsinki.fi (2007).

[52] Guler HP, Zapf J, Scheiwiller E, Froesch ER (1988). Recombinant human insulin-like growth factor I stimulates growth and has distinct effects on organ size in hypophysectomized rats. Proc. Natl. Acad. Sci. USA.

[53] Zhao G (2000). Targeted overexpression of insulin-like growth factor I to osteoblasts of transgenic mice: Increased trabecular bone volume without increased osteoblast proliferation. Endocrinology.

[54] Oshima, M. *et al*. Functional tooth regeneration using a bioengineered tooth unit as a mature organ replacement regenerative therapy. *PLoS One* **6**, 10.1371/journal.pone.0021531. (2011). 10.1371/journal.pone.0021531 PMC3134195 21765896

