# Supplementary material for: Insulin-like growth factor 1 modulates bioengineered tooth morphogenesis
Source: Sci Rep. 2019 Jan 23;9:368. doi: 10.1038/s41598-018-36863-6 (PMC6344556; doi:10.1038/s41598-018-36863-6)
Supplement: Supplementary file 1 — Supplementary Information [file 41598_2018_36863_MOESM1_ESM.pdf]

Insulin-like growth factor 1 modulates bioengineered tooth morphogenesis

Toshihito Oyanagi[1]*, Nobuo Takeshita[1]*, Mamiko Hara[1]*, Etsuko Ikeda[1, 2], Toko Chida[1], Daisuke Seki[1], Michiko Yoshida[1], Masahiro Seiryu[1], Ikuko Takano[1], Seiji Kimura[1], Masamitsu Oshima[3], Takashi Tsuji[2], Teruko Takano-Yamamoto[1, 4]**

[1]Division of Orthodontics and Dentofacial Orthopedics, Graduate School of Dentistry, Tohoku University, Sendai, Miyagi, 980-0875, Japan.

[2]Laboratory for Organ Regeneration, RIKEN Center for Developmental Biology, Kobe, Hyogo, 650-0047, Japan.

[3]Department of Stomatognathic Function and Occlusal Reconstruction, Graduate School of Biomedical Sciences, Tokushima University, Tokushima, Tokushima, 770-8501, Japan.

[4]Department of Biomaterials and Bioengineering, Faculty of Dental Medicine, Hokkaido University, Sapporo, Hokkaido, 060-8586, Japan.

*These authors contributed equally to this work.

**Corresponding author : Teruko Takano-Yamamoto, DDS, PhD, Division of Orthodontics and Dentofacial Orthopedics, Tohoku University Graduate School of Dentistry, 4-1, Seiryomachi, Aoba-ku, Sendai, 980-8575, Japan

Tel : +81 22 7178374, Fax : +81 22 7178378, E-mail : t-yamamo@m.tohoku.ac.jp

Supplementary Information

Supplementary Methods

Measurement of the size of tooth germs and teeth

Tooth germs were photographed at day 7 and 14 of organ culture using a phase contrast microscope (IX71; Olympus, Tokyo, Japan), and the length and width of tooth germs were measured using image analysis software, Cell Sens (Olympus) (Supplementary Fig. S1a). We first drew a line (line a) connecting cusp tips, and then drew another line (line b) perpendicular to line a. Next, we drew another line (line c) perpendicular to line b through the lowest point of a tooth germ at cervical side. The height of tooth germs was defined as the length between line a and c. We drew a fourth line (line d) perpendicular to line b to measure the maximum width of the tooth germ.

We measured the length and width of the bioengineered teeth developed in subrenal capsules and jawbones on micro-CT images using i-VIEW-3DX (Supplementary Fig. S1b). The height (e) of teeth was defined as the maximum length from the cusp tip to the apex along the long axis of tooth. The width (f) of a tooth was defined as the maximum length of the crown on the horizontal plane being perpendicular to the long axis of tooth.

Gene Name	Primer Sequence	Accession No.
Fgf4	5'-TGACATCAGTTTCCAAGGGTGC-3' (S)<br>5'-CCAGAGTTTTGAGTTGTGGCGAG-3' (AS)	NM_010202

Supplementary Table S1. Primers used to amplify DNA fragment of *Fgf4*.

Gene Name	Primer Sequence	Accession No.
Pitx2	5'-ATGTTTTCCCCGCCCAACTC-3' (S)<br>5'-TTCAGGCTATTGAGGCTGGAGC-3' (AS)	NM_011098
Shh	5'-ACATTGGCACCTGGCTGTTG-3' (S)<br>5'-AGTTCCTTATTTCGCAGTCGCTC-3' (AS)	NM_009170
Fgf4	5'-TCCTTTCTGTCTGGTGGCTCAC-3' (S)<br>5'-ACTGTCTTCTCCCCAACACTCTTG-3' (AS)	NM_010202
Fgf10	5'-CAGCCCCAAACAACAACAACAG-3' (S)<br>5'-TGAGTAGTTAGGAGAACCCGAAAGC-3' (AS)	NM_008002
Ambn	5'-GGGACTCAACGCACTTTCTCAG-3' (S)<br>5'-AAGGAGGCTGCTGGGTTTC-3' (AS)	NM_001303431
Dmp1	5'-ACCACACGGACAGCAGTGAATC-3' (S)<br>5'-CCTCATCGCCAAAGGTATCATCTC-3' (AS)	NM_016779
Dspp	5'-CGACGAATCTAACGGAAGTGACG-3' (S)<br>5'-TGTAAGAAGCATCTCCACGGCTG-3' (AS)	NM_010080
Map1b	5'-CCTCTCCTACCTGTGTGGCTAATC-3' (S)<br>5'-CCATTGTGGTGGCTACCTCTAAAC-3' (AS)	NM_008634
Igf1	5'-CTGAGCTGGTGGATGCTCTTC-3'(S)<br>5'-CACTCATCCACAATGCCTGTCT-3'(AS)	NM_001314010
Igf1r	5'-CATTTGTGGGTTTGGTTTGGTC-3'(S)<br>5'-CATAGGAAGCAGACAGGGGTTG-3'(AS)	NM_010513
Bmp2	5'-CAGAGTGGTTGTCCAATCCGTG-3'(S)<br>5'-TGGTCCTGCTTTGTTCTTGCTG-3'(AS)	NM_007553
Igfbp2	5'-CCCGAGCCATCTCTTCTACAAC-3'(S)<br>5'-CCCTCCATTTCTGCTGGTGTTC-3'(AS)	NM_008342
Igfbp3	5'-TCTACTTGGGAACTGTGGGAGAGG-3'(S)<br>5'-CCTTATCCTTGTCCTTATGGGCTC-3'(AS)	NM_008343
Igfbp4	5'-TGACGAGGTGTTCTCAACTCAAGG-3'(S)<br>5'-AGGGAGGGCGTTCAATGTTCAC-3'(AS)	NM_010517
Igfbp6	5'-CTATGTGCCAAACTGTGACCTCAG-3'(S)<br>5'-GTGCTTCCTTGACCATCTGGAG-3'(AS)	NM_008344
Gapdh	5'-TGTGTCCGTCGTGGATCTGA-3' (S)<br>5'-TTGCTGTTGAAGTCGCAGGAG-3' (AS)	NM_001289726

Supplementary Table S2. Primers used for RT-PCR and real-time PCR.

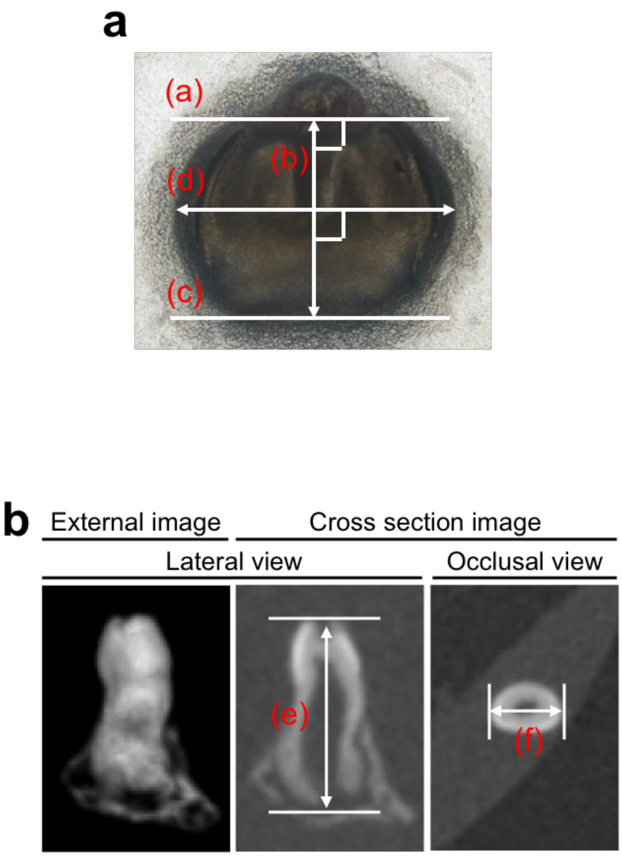

Supplementary Figure S1. Measurement of the size of tooth germs and teeth. (**a**) Phase contrast images of bioengineered tooth germs. (**b**) Micro-CT images of bioengineered teeth. Details about the measurement of the size of tooth germs and teeth were described in the Supplementary Methods.

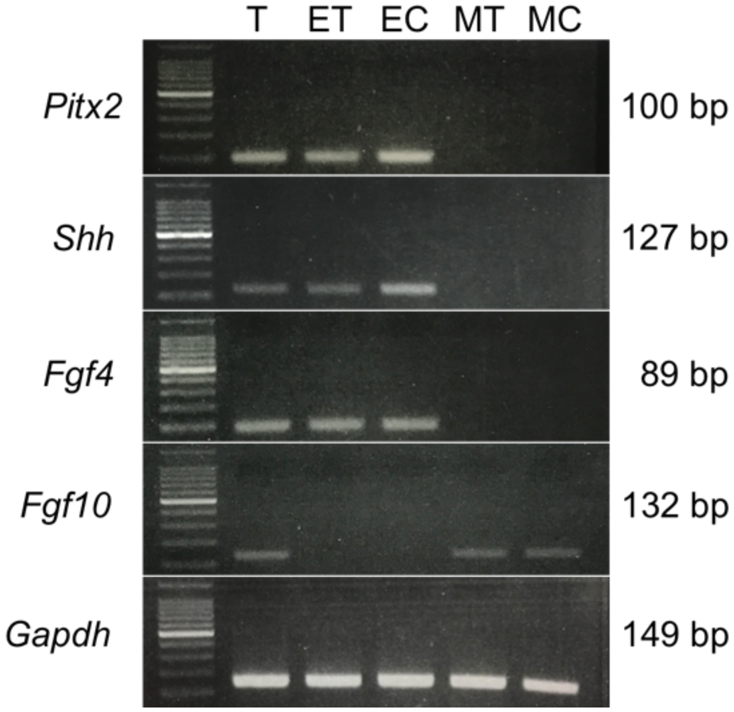

Supplementary Figure S2. Analysis of gene expressions in epithelial and mesenchymal cells isolated from the ED 14.5 mouse mandibular molar tooth germs. The mRNA expressions of *Pitx2*, *Shh*, *Fgf4, Fgf10* and *Gapdh* were analysed by RT-PCR. The product size of the amplified cDNA was *Pitx2*: 100 bp, *Shh*: 127 bp, *Fgf4*: 89 bp, *Fgf10*: 132 bp, *Gapdh*: 149 bp. T, tooth germ; ET, dental epithelial tissue; EC, dental epithelial cell; MT, dental mesenchymal tissue; MC, dental mesenchymal cell

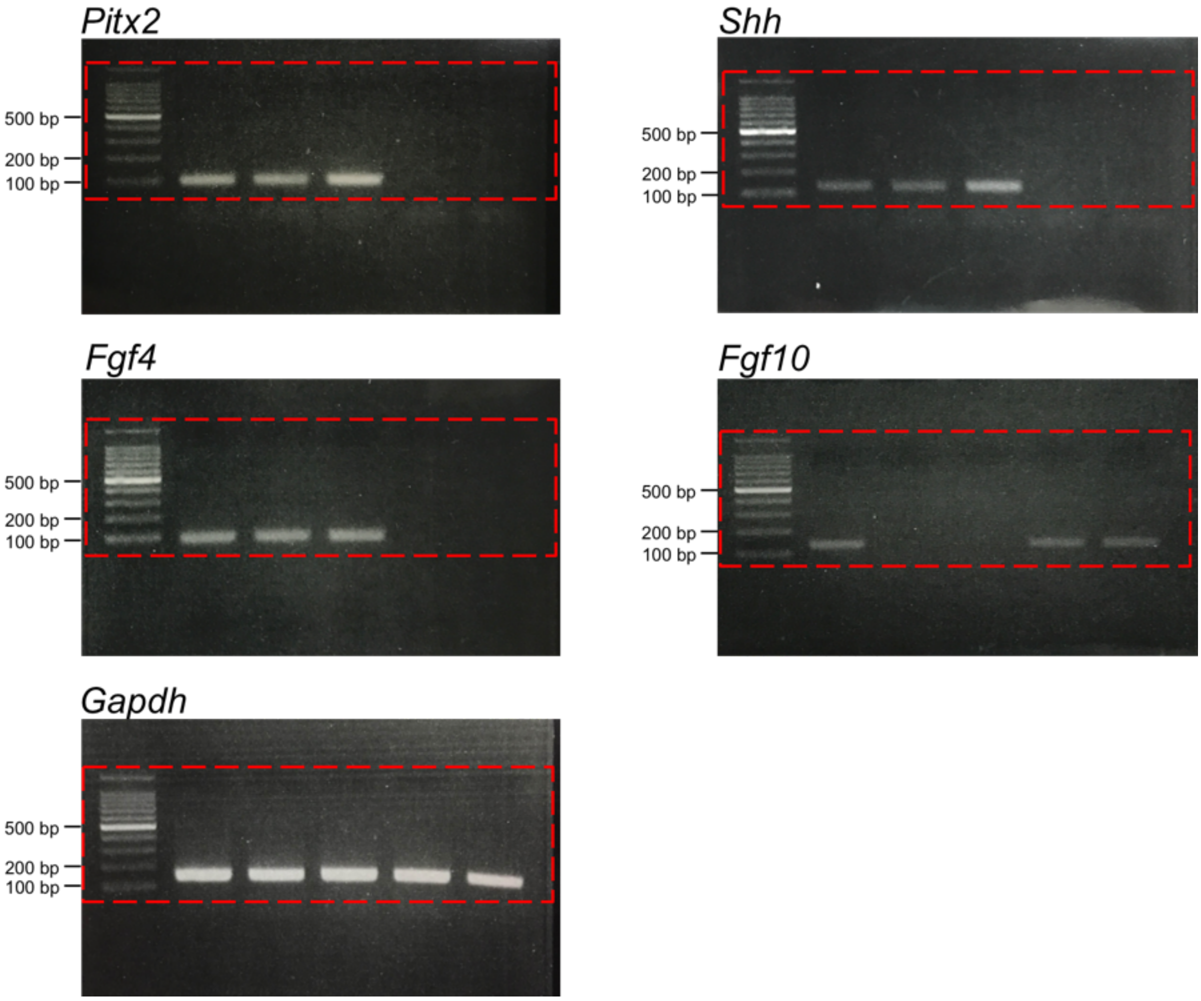

Supplementary Figure S3. Original photos corresponding to Supplementary Figure S2.

Dotted red lines show the cropped region.

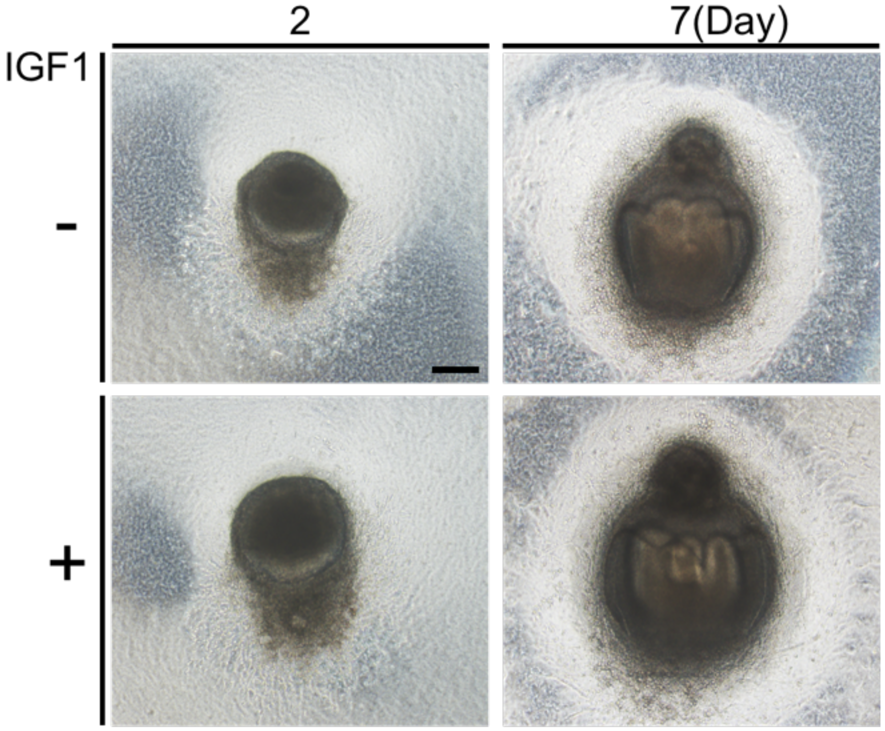

Supplementary Figure S4. Phase contrast images of bioengineered tooth germs at days 2 and 7 of organ culture in the control and IGF1-treated groups. Scale bar, 200 μm.

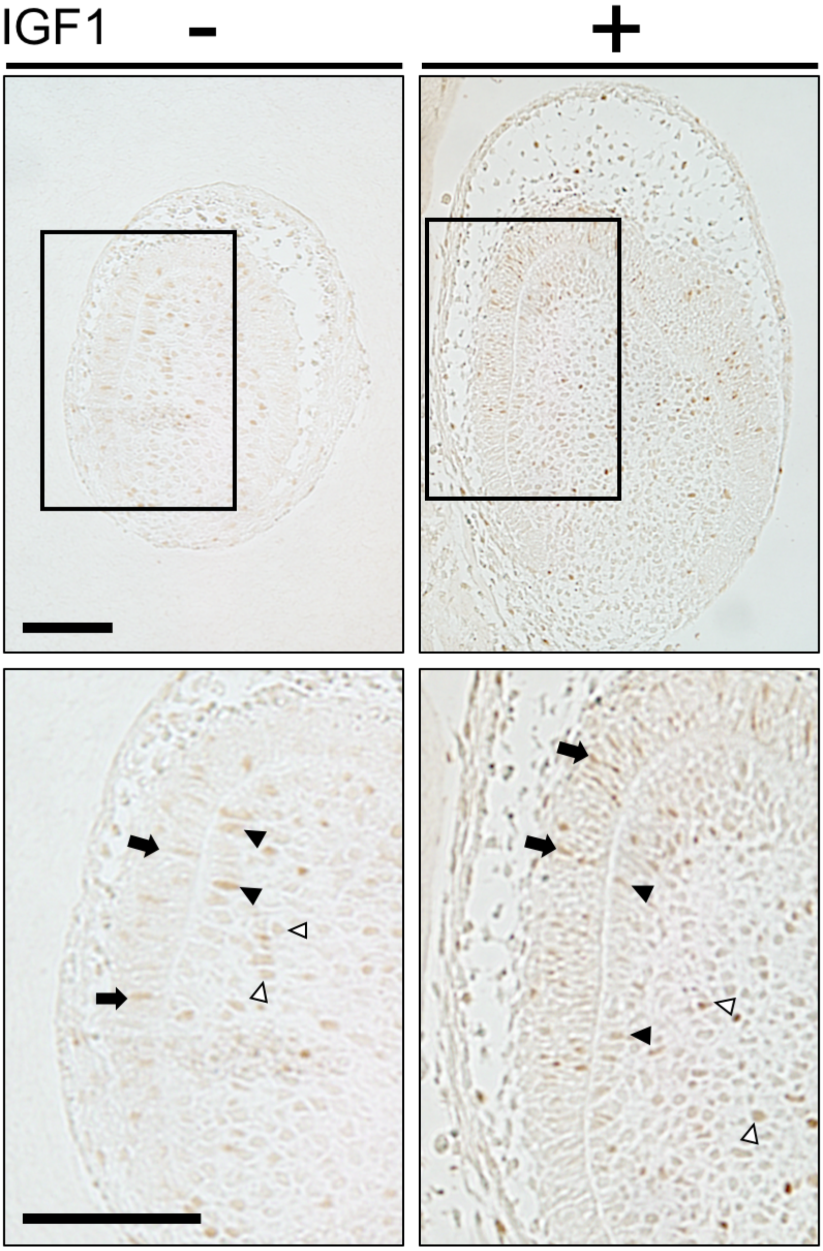

Supplementary Figure S5. Immunohistochemical staining of IGF1R in bioengineered tooth germs at day 7 of culture in the control and IGF1-treated groups. IGF1R was expressed in ameloblasts (black arrows), odontoblasts (black arrowheads) and dental papilla cells (white arrowheads). The boxed areas in the upper panels are shown as enlarged images in the lower panels. Scale bar, 100 µm.

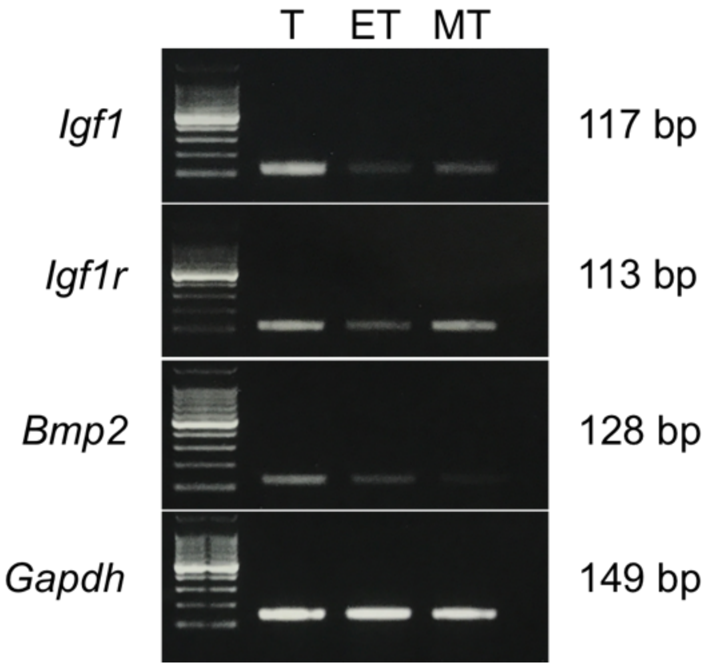

Supplementary Figure S6. Analysis of gene expressions in epithelial and mesenchymal tissues isolated from the ED 14.5 mouse mandibular molar tooth germs. The mRNA expressions of *Igf1*, *Igf1r*, *Bmp2* and *Gapdh* were analysed by RT-PCR. The product size of the amplified cDNA was *Igf1*: 117 bp, *Igf1r*: 113 bp, *Bmp2*: 128 bp, *Gapdh*: 149 bp. T, tooth germ; ET, dental epithelial tissue; MT, dental mesenchymal tissue

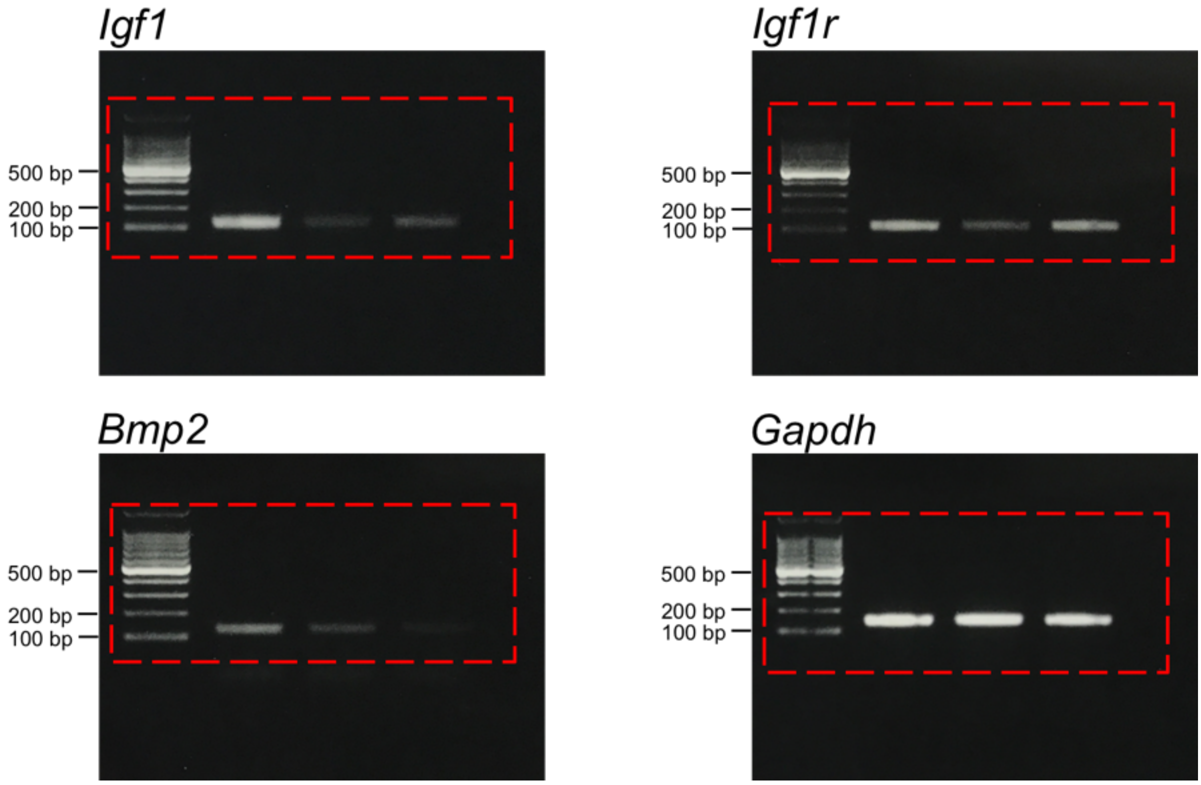

Supplementary Figure S7. Original photos corresponding to Supplementary Figure S4.

Dotted red lines show the cropped region.
